# A multiplex platform for small RNA sequencing elucidates multifaceted tRNA stress response and translational regulation

**DOI:** 10.1038/s41467-022-30261-3

**Published:** 2022-05-05

**Authors:** Christopher P. Watkins, Wen Zhang, Adam C. Wylder, Christopher D. Katanski, Tao Pan

**Affiliations:** 1 Department of Biochemistry and Molecular Biology, Chicago, IL 60637 USA; 2 Department of Chemistry, University of Chicago, Chicago, IL 60637 USA; 3 Department of Molecular Genetics and Cell Biology, University of Chicago, Chicago, IL 60637 USA

**Keywords:** RNA sequencing, tRNAs

## Abstract

Small RNAs include tRNA, snRNA, micro-RNA, tRNA fragments and others that constitute > 90% of RNA copy numbers in a human cell and perform many essential functions. Popular small RNA-seq strategies limit the insights into coordinated small RNA response to cellular stress. Small RNA-seq also lacks multiplexing capabilities. Here, we report a multiplex small RNA-seq library preparation method (MSR-seq) to investigate cellular small RNA and mRNA response to heat shock, hydrogen peroxide, and arsenite stress. Comparing stress-induced changes of total cellular RNA and polysome-associated RNA, we identify a coordinated tRNA response that involves polysome-specific tRNA abundance and synergistic N3-methylcytosine (m^3^C) tRNA modification. Combining tRNA and mRNA response to stress we reveal a mechanism of stress-induced down-regulation in translational elongation. We also find that native tRNA molecules lacking several modifications are biased reservoirs for the biogenesis of tRNA fragments. Our results demonstrate the importance of simultaneous investigation of small RNAs and their modifications in response to varying biological conditions.

## Abstract (web summary)

This paper develops a multiplex RNA-seq method that reports tRNA abundance, modification, charging, and fragmentation. Results show stress-induced regulation in translational elongation and association of modification and tRNA fragment biogenesis.

## Introduction

High-throughput RNA sequencing has provided transformative insights into cellular homeostasis, dynamic response to stress and environmental change, and RNA modifications. However, significant challenges associated with the characterization of small RNAs remain. Small RNAs are less than 200 nucleotides in length and include transfer RNAs (tRNA), microRNAs, small nucleolar RNAs, tRNA fragments, and many others that play important roles in cellular pathways and physiology. Altogether, small RNAs constitute more than 90% of cellular RNAs in copy numbers; among these, tRNA is the most abundant^1^. The role of tRNA in translational regulation depends on the expression and aminoacylation (charging) levels of different tRNA species, as well as many modifications that fine-tune tRNA activity^2–4^. Therefore, comprehensive and high-throughput characterization of tRNA is essential for a deeper understanding of the biological function of small RNAs.

Most commonly used RNA-seq methods are incompatible with the comprehensive study of small RNAs. Many small RNA-seq techniques ligate adapter oligonucleotides to the target RNAs, followed by cDNA synthesis. Products of incomplete reverse transcription, often induced by RNA modification or structure, are not amplified and not included in downstream analysis. tRNA is the RNA family most limited by these methods, due to rigid secondary structure and extensive modification. Several recent approaches have profoundly advanced our ability for efficient and quantitative tRNA sequencing^5–11^. Cozen et al. (ARM-seq)^6^ and Zheng et al. (DM-tRNA-seq)^5^ both used *E. coli* AlkB demethylases to remove the abundant Watson–Crick face methylations in tRNA before the reverse transcriptase (RT) reaction. Pinkard et al (QuantM-tRNA-seq)^7^ used 3′CCA specific adapter ligation that specifically tagged tRNA for improved library construction. DM-tRNA-seq also used a thermostable RT (TGIRT) to facilitate a read-through of tRNA modifications and secondary structure. Behrens et al. (mim-tRNA-seq)^8^ found a TGIRT reaction condition that significantly improved the yield of cDNA synthesis of full-length tRNA. Gogakos et al. (Hydro-tRNA-seq)^10^ carried out partial alkaline hydrolysis first which reduced the structural context of tRNA to improve the efficiency of tRNA library construction. Hu et al. (AQRNA-seq)^11^ used a one-pot reaction in library construction including the removal of excess adapter oligos by a nuclease. A common drawback of these approaches involves size selection steps or sequence context requirements which limit the investigation of RNAs to a certain size range or to specific families and uncouples the coordinated expression and response of small RNA families, e.g., tRNA with tRNA fragments. Finally, small RNA-seq procedures also lack the level of multiplexing enjoyed by mRNA sequencing^12^. Therefore, new small RNA-seq methods are still needed to better characterize tRNA properties, incorporate tRNA results with other small RNA families, and increase multiplexing capability.

Here, we describe **m**ultiplex **s**mall RNA sequencing (MSR-seq), a platform for RNA-seq library construction that provides multiplexing to greatly increase throughput. The key feature of MSR-seq is the design of a biotinylated oligonucleotide that is used for barcode adapter ligation, immobilization, on-bead reverse transcription, second adapter ligation, and PCR. This unification of multiple steps in RNA-seq library construction enables multiplexing of many samples in the same reaction which increases sample handling throughput and reduces input amount. Our method also allows for the inclusion of enzymatic and chemical treatment of RNA on-bead, thus accommodating the investigation of RNA modifications and other applications. For biological application, we investigated the stress response of tRNA abundance, charging, and modification, as well as other small RNA families upon heat shock, exposure to hydrogen peroxide, and to arsenite which are known to strongly affect translation^13–15^. Using MSR-seq measurements of tRNA in total RNA and in the polysome, we identify specific tRNA responses. Together with mRNA measurements, the tRNA response is consistent with stress- and tRNA-dependent translational downregulation during translational elongation. We also find native tRNA molecules lacking several modifications as altered reservoirs for tRNA fragment biogenesis.

## Results

### MSR-seq immobilizes RNA for multiplexed library construction

**Fig. 1:**
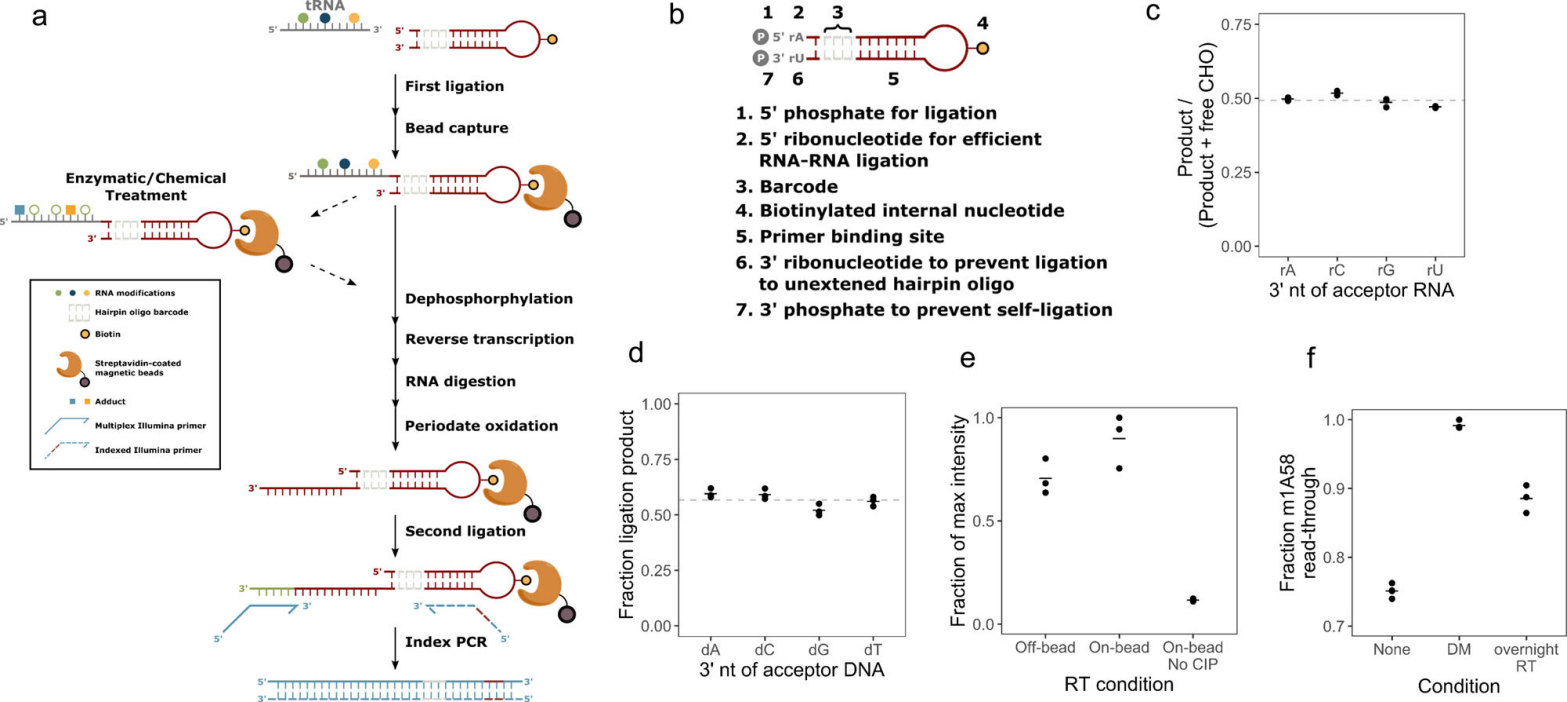
Design and optimization of MSR-seq. **a** Schematic representation of multiplex small RNA-seq. The required steps are indicated in solid arrows and optional steps in dashed arrows. Symbols are explained in the box. **b** Features of the capture hairpin oligo (CHO) with detailed descriptions for each feature. **c** Fraction of ligation products for the test RNA oligonucleotides containing rA, rC, rG, and rU at the 3′ end. *n* = 3 independent experiments. Quantitative data were from denaturing gels stained with SYBR Gold shown in Supplementary Fig. 1a. The mean value is shown as a bar; the mean of all replicates is shown as a dashed line. The molar ratio as measured by UV absorbance of input RNA and the CHO in the ligation reaction was ~1:2. Fraction product is calculated as (Product)/(Product + Free CHO). The expected stoichiometric amount of ligation product corresponds to ~0.5. **d** Fraction of ligation products for the test 5′ biotinylated deoxyoligonucleotides containing dA, dC, dG, and T at the 3′ end. *n* = 3 independent experiments. Quantitative data were from denaturing gels stained with SYBR Gold shown in Supplementary Fig. 1b. The mean value is shown as a bar; the mean of all replicates is shown as a dashed line. Fraction product is calculated as (Product)/(Product + Free 5′biotin-oligo). **e** Relative reverse transcription products with RT performed free in solution (off-bead), on streptavidin bead (on-bead), and on-bead without calf-intestine alkaline phosphatase treatment (No-CIP). Input samples were total RNA from HEK293T cells. *n* = 3 independent experiments. Quantitative data were from native gels stained with SYBR Gold shown in Supplementary Fig. 1c. The mean value of replicates is shown as a bar. Product is defined as all bands above the terminal transferase product of the RT (TdT) band in Supplementary Fig. 1c. Fraction product is calculated as (Product in each sample)/(sample with the maximum amount of product). The non-zero value from No-CIP samples was from spurious oligos associated with the beads, see Supplementary Fig. 1c. **f** Fraction of reverse transcriptase m^1^A58 read-through product with no demethylase treatment/short RT reaction (none), demethylase treatment/short RT reaction (DM), and no demethylase treatment/long RT reaction (overnight RT). Input samples were total RNA from HEK293T cells. *n* = 3–4 independent experiments. Quantitative data were from native gels stained with SYBR Gold shown in Supplementary Fig. 1e, h. The mean value of replicates is shown as a bar. Product is defined as all bands above the m^1^A58 band in Supplementary Fig. 1e, h. Product is defined as all bands above the TdT band in Supplementary Fig. 1c. Fraction product is calculated as (Product)/(Product + m^1^A58 stopband).

The basic steps of small RNA-seq library construction include first adapter ligation, reverse transcription, second adapter ligation, and PCR amplification. Additions can include enzymatic or chemical treatments of the RNA to profile RNA modifications or map RNA structures. Our goal is to immobilize and barcode RNA samples at the earliest step possible, thereby enabling all subsequent reactions, including optional enzymatic/chemical reactions to proceed on a solid support, which increases multiplexing potential and minimizes sample handling (Fig. 1a). First, a barcode was ligated to the 3′ of input RNA from any biological source. Since >90% of cellular RNA in copy numbers were small RNA, they constituted most of the ligated product, thus further isolation was not necessary. Barcoded samples were pooled and immobilized on streptavidin beads. Enzymatic or chemical treatments could then be added before reverse transcription. On-bead reverse transcription was followed by second adapter ligation to the cDNA and PCR; the off-bead PCR products were readily used for sequencing.

We designed a capture hairpin oligonucleotide (CHO) that contains several features to make all steps compatible with streptavidin beads (Fig. 1b). The salient features include: (i, ii) a 5′ phosphate and ribonucleotide for efficient first ligation;^16^ (iii) a barcode sequence for sample pooling and multiplexing; (iv) a biotin moiety for immobilization; (v) a primer binding site embedded in the hairpin for reverse transcription and PCR; (vi) a 3′ ribonucleotide to prevent second adapter ligation to free CHO: this ribonucleotide enables periodate oxidation of the unligated CHO which converts its 2′,3′ hydroxyls to 2, 3′ aldehydes at the 3′ end. Only 2′,3′ hydroxyls allow for the ligation of the second adapter. (vii) a 3′ phosphate to prevent self-ligation of the excess CHO.

We optimized reaction conditions and demonstrate high efficiency and equity for RNA substrates^17^. To examine the bias of the 3′ end nucleotide identity of the sample RNA in the first ligation, we measured the ligation efficiency of four RNA oligonucleotides of 5′ N_10_X (X = A, C, G, U) as the input samples. The ligation generated stoichiometric amounts of products for all four RNA oligos under our reaction condition (Fig. 1c and Supplementary Fig. 1a). To examine the bias of the 3′ end nucleotide identity of cDNA products in the second ligation, we used four 5′ biotinylated DNA oligonucleotides ending with dN_10_dX (X = dA, dC, dG, T) as the input samples. The on-bead ligation generated high levels of product with little bias for the 3′ deoxynucleotide identity (Fig. 1d and Supplementary Fig. 1b). To examine the efficiency of on-bead reverse transcription, we used total HEK293T RNA as input and ran the RT reaction in parallel either on or off the streptavidin bead and measured the product amounts by RT-PCR. The on-bead RT reaction generated a higher amount of products than the off-bead RT reaction (Fig. 1e and Supplementary Fig. 1c).

We deployed two innovations to enable all steps after the first barcode ligation to be performed on-bead. First, we added a 3′ phosphate that blocked the self-ligation of CHO during the first ligation. This phosphate was removed on-bead to allow for subsequent reverse transcription from the 3′ OH. No specific product was visible without the 3′ phosphate removal by phosphatase (Fig. 1e and Supplementary Fig. 1c). Second, we designed a 3′ ribonucleotide in CHO which enabled the reduction of PCR products from the excess CHO on-bead. After cDNA synthesis, the CHO with ligated input RNA contained a terminal deoxyribonucleotide which protected it from periodate oxidation and thus made it a substrate for the second adapter ligation. In contrast, the excess, free CHO containing 3′ ribonucleotide was periodate oxidized and no longer a ligation substrate (Supplementary Fig. 1d).

After the first ligation and streptavidin bead binding of all CHO, the sample could be optionally split in two: one mock-treated, and the other subjected to chemical or enzyme treatment. To validate that enzyme treatment is compatible with bead immobilization, we compared libraries prepared with and without AlkB treatment. The on-bead AlkB demethylase reaction efficiently reduced the PCR product bands derived from tRNA m^1^A/m^1^G modification relative to the full-length tRNA^5^ (Fig. 1f and Supplementary Fig. 1e). To facilitate tRNA charging studies^17^, we modified the oxidation and β-elimination protocol to enable the sequential addition of these reagents in a single tube. The product of this β-elimination reaction is used directly in the CHO ligation so that no reaction intermediates were precipitated or purified (Supplementary Fig. 1f, g).

The only caveat of our approach was the known terminal deoxyribonucleotide transferase activity (TdT) of reverse transcriptases^18^, which produced varying amounts of PCR products derived from the free CHO on-bead. This artificial product could be removed through gel extraction of the final PCR products.

Although demethylase treatment in our DM-tRNA-seq method improved efficiency and quantitation^5^, it results in duplication of library preparation for the same sample, rendering it less practical for high volume tRNA-seq experiments. Similar to mim-tRNA-seq^8^, we found an SSIV RT reaction condition (Fig. 1f and Supplementary Fig. 1h) that could more effectively read through tRNA methylations, thus enabling the investigation of tRNA abundance, charging, and modification simultaneously with a single sequencing library.

**Fig. 2:**
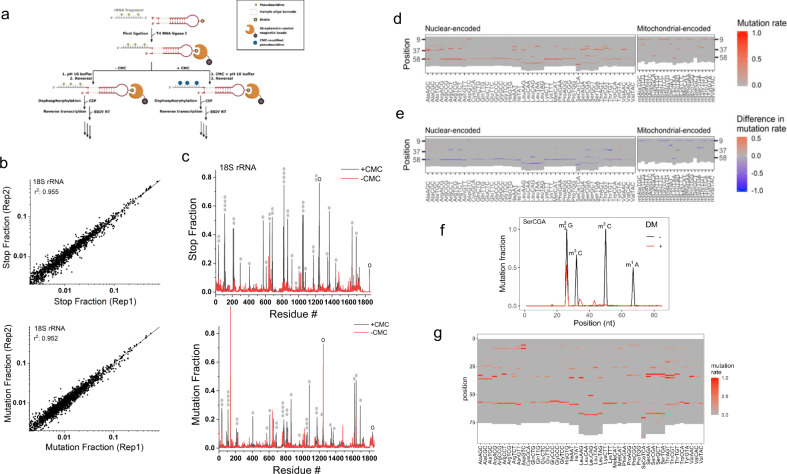
Sequencing results from chemical and enzymatic treatment on bead. **a** Schematic representation of incorporating CMC reaction in MSR-seq for pseudouridine (Ψ) mapping. Total RNA was fragmented, 3′ end-repaired, and ligated to the capture hairpin oligonucleotide. CMC reaction was done on-bead before cDNA synthesis. **b** CMC reaction mapping replicates: Top graph shows the RT stop fraction and the bottom graph shows the mutation fraction for every residue in human 18 S rRNA among the biological replicates. **c** Stop and mutation fraction along 18 S rRNA without (red) and with CMC (black) treatment: Top graph shows the RT stop fraction and the bottom graph shows the mutation fraction at each nucleotide position. Known Ψ sites are marked by filled gray ovals, known m^1^acp^3^Ψ and m^6^_2_A sites by open ovals. **d** Heat map showing the mutation signature at every nucleotide for the most abundant tRNA isodecoder in each isoacceptor family, no demethylase treatment. Nuclear-encoded and mitochondrial-encoded tRNAs are shown separately. **e** Heat map showing changes in mutation signature upon demethylase (DM) treatment at every nucleotide for the most abundant tRNA isodecoder in each isoacceptor family. Red indicates an increase and blue indicates a decrease in mutation fraction. **f** Mutation fraction across a tRNA^Ser^(CGA) isodecoder ± demethylase treatment (DM) showing the effective removal of base methylation. Known modifications are indicated. **g** Same as (**d**), using overnight RT condition for library construction.

MSR-seq also allows for chemical treatment of RNA on-bead which is useful for RNA modification studies or RNA structural mapping^19^. We used the well-established *N*-cyclohexyl-*N*′-β-(4-methylmorpholinium) ethylcarbodiimide (CMC) reaction for pseudouridine (Ψ) modification^20^ to test the compatibility of MSR-seq on-bead protocol with harsh chemical treatment conditions (Fig. 2a). To map the Ψ sites in human rRNA, we fragmented total RNA, ligated the fragments to the CHO, then performed the CMC reaction on-bead. We assigned each rRNA position a stop and mutation fraction and observed a good correlation between biological replicates (Fig. 2b and Supplementary Fig. 2a). We identified strong signals in the stop and/or mutation fractions in the CMC-treated sample at the 35 of 36 known Ψ sites^21^ in the 18 S rRNA (Fig. 2c), validating the usefulness of our approach.

To quantitatively compare the MSR-seq result with another previously published tRNA-seq method, we compared MSR-seq and DM-tRNA-seq results for tRNA isodecoder expression in HEK293T cells^5^. We observed a good correlation (Supplementary Fig. 2b), even though MSR-seq and DM-tRNA-seq used different RTs, steps in library construction, and input RNAs. To test the sample input limits of the MSR-seq method, we built libraries starting with 1000, 100, and 10 ng of total HEK293T RNA (Supplementary Fig. 2c). tRNA abundance was well correlated between these libraries with *r*^2^ ~0.94.

To validate that demethylase treatment worked well on bead, we found that Watson–Crick face methylations such as m^1^A, m^3^C, and m^1^G produced substantial mutation signatures as expected from previous studies^22,23^ (Fig. 2d), but demethylase treatment abolished or reduced the mutation fractions associated with these methylations (Fig. 2e, f). Validation of mutation signature as RNA modifications with demethylase treatment still has value when working with less well-characterized samples. We also found that our overnight RT condition reports a very similar tRNA modification landscape as the short RT reaction (Fig. 2g).

### Stress induces coordinated tRNA abundance and modification changes in total RNA

**Fig. 3:**
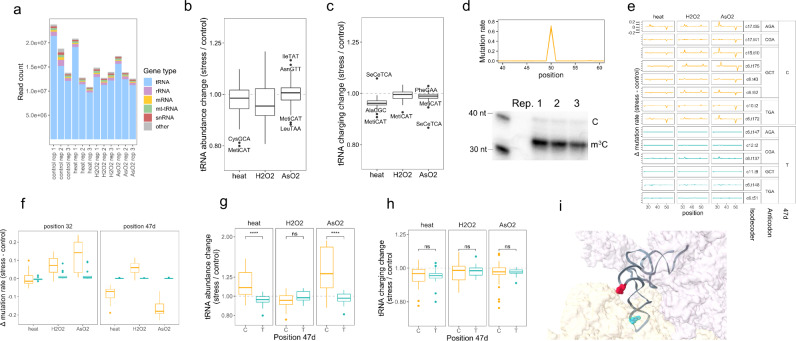
tRNA analysis in total RNA under stress. MSR-seq was performed in biological triplicates (*n* = 3) for each condition using HEK293T cells: unstressed, 42 °C/1 h (heat), 0.6 mM H_2_O_2_/2 h (H_2_O_2_), 0.3 mM NaAsO_2_/2 h (AsO_2_). Box and Whisker plots show median, 25th and 75th quartile, and whiskers to 1.5 times interquartile range. **a** Read coverage among RNA families for each sample. **b** Stress-induced tRNA abundance change among isoacceptor families. Abundance is calculated as summed normalized coverage of isodecoders in an isoacceptor family. The abundance change upon stress is calculated for each isoacceptor family, averaged across *n* = 3 independent biological replicates, displaying 46 isoacceptor families. **c** Stress-induced tRNA charging change among isoacceptor families. Charging is calculated as the ratio of the sum of charged isodecoder reads (CCA-ending) to the sum of uncharged isodecoder reads (CC-ending) for an isoacceptor family. The change in this ratio between stressed and unstressed samples is calculated for each isoacceptor family, then averaged across *n* = 3 independent biological replicates, displaying 46 isoacceptor families. **d** Top graph shows the mutation fraction at the 50th nucleotide position in tRNA^Ser^(GCT) as a representative isodecoder; this position corresponds to 47d in tRNA nomenclature. The three independent biological replicates are shown. The bottom gel image shows validation of sequencing mutation fractions of m^3^C47d in tRNA^Ser^(GCT) by primer extension: the shorter product indicates an m^3^C-induced RT stop, while the longer product indicates read through of hypomodified molecules. Source data are provided as a Source Data file. **e** The difference in mutation fraction between stressed and unstressed samples is plotted for tRNA^Ser^ isodecoders, highlighting differences in m^3^C32 and m^3^C47d mutation signature (*n* = 3 replicates shown). C47d-tRNA^Ser^ isodecoders are in orange, T47d-tRNA^Ser^ in blue. Isodecoder designations are according to the hg19 genomic tRNA database^59^, e.g., c17.t35 corresponds to chromosome 17, tRNA35. Change in mutation fraction for each replicate is calculated as mutation fraction minus the mean of control replicates. **f** Summary of data from panel **e**: mutation fraction at sites corresponding to tRNA^Ser^ m^3^C32 and m^3^C47d change under stress. C47d-tRNA^Ser^ isodecoders are in orange and T47d-tRNA^Ser^ in blue (each point is a tRNA^Ser^ isodecoder). The mutation fraction at T47d is zero; these sites are not methylated. Data were shown for *n* = 3 independent biological replicates of 8 and 6 C or T tRNA^Ser^ isodecoders, respectively. **g** Abundance change for C47d-tRNA^Ser^ isodecoders in orange and T47d-tRNA^Ser^ in blue. ****p < 10^−4^, ns not significant; significance calculated with two-sided Wilcoxon test. Data were shown for *n* = 3 independent biological replicates of 8 and 6 C or T tRNA^Ser^ isodecoders, respectively. *P* values are 1.2 × 10^−5^ and 1.3 × 10^−5^ for heat and arsenite stress. **h** Charging change for C47d-tRNA^Ser^ isodecoders in orange and T47d-tRNA^Ser^ in blue. ns not significant; significance calculated with two-sided Wilcoxon test. Data were shown for *n* = 3 independent biological replicates of 8 and 6 C or T tRNA^Ser^ isodecoders, respectively. **i** Location of m^3^C32 in cyan and m^3^C47d in red in the 3D structure of ribosome-bound tRNA^Ser^ (PDB 6Z6M from ref. ^25^). The 40 S subunit is in light yellow, 60 S subunit is in light purple.

We applied MSR-seq to investigate the stress response of tRNAs. To broaden the scope of our study, we subjected HEK293T cells to three commonly used, but different stress types: heat shock, hydrogen peroxide, and arsenite, plus the unstressed control. Using total RNA as input, we obtained on average 15 million reads mapped to the human genome among all samples (Fig. 3a). As expected, most reads were from nuclear-encoded tRNA, with the remaining from 5 S and 5.8 S rRNA, mitochondrial-encoded tRNA, spliceosomal RNA (snRNA), mRNA, and other RNA families.

We analyzed the nuclear-encoded tRNA results by comparing each stress condition with unstressed control. We examined tRNA abundance, charging, and modification at the isoacceptor level wherein we pooled reads mapped to tRNA genes with the same anticodon. At the isoacceptor level, tRNA abundance changes were within 1.25-fold (Fig. 3b and Supplementary Data 1), and tRNA charging level within 1.15-fold (Fig. 3c and Supplementary Data 2), indicating that tRNA abundance and charging in total cellular tRNA did not change widely under these stress conditions.

We searched for tRNA isodecoder modification responses under stress and identified m^3^C in serine tRNAs. Using primer extension, we validated that the mutation fraction of m^3^C in MSR-seq could be used to quantify the modification fraction (Fig. 3d). In human tRNAs, m^3^C is present in the anticodon loop of tRNA^Arg^(yCU)/tRNA^Ser^/tRNA^Thr^ (m^3^C32 in tRNA nomenclature) and in the loop of the variable arm stem-loop of tRNA^Leu^(CAG)/tRNA^Ser^ (m^3^C47d or m^3^Ce2 in tRNA nomenclature)^23,24^. The mutation fraction of m^3^C47d in tRNA^Ser^, but not in tRNA^Leu^(CAG) showed a marked decrease under heat and arsenite stress, and an increase under hydrogen peroxide stress (Fig. 3e).

Human tRNA^Ser^ isodecoder genes have either C47d or T47d (Supplementary Fig. 3). Isodecoders with T47d cannot be methylated and no mutation signature was observed. During stress, the m^3^C32 for the C47d-tRNA^Ser^ isodecoders generally showed an increase in the mutation fraction, whereas the T47d isodecoders showed little change (Fig. 3e, f). During heat and arsenite stress only C47d-tRNA^Ser^ isodecoders showed an increase in abundance, but T47d-tRNA^Ser^ isodecoders showed little change (Fig. 3g). By contrast, there was no significant change in the charging levels of either sets of isodecoders under stress (Fig. 3h). m^3^C introduces a positive charge at physiological pH. In the three-dimensional structure of tRNA^Ser^ on the ribosome, m^3^C32 is close to the tRNA-mRNA base pairs in the 40 S, and m^3^C47d is at the 40S–60S interface^25^ (Fig. 3i). These results suggest that m^3^C47d and m^3^C32 modifications can respond synergistically under stress.

### Stress induces coordinated tRNA abundance and modification changes in polysome

**Fig. 4:**
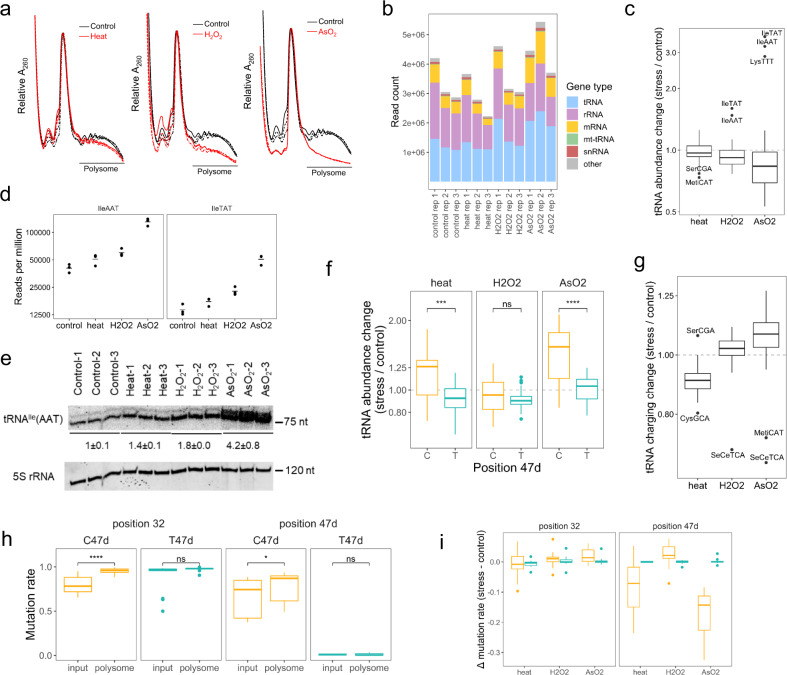
tRNA analysis of polysome under stress. MSR-seq was performed in biological triplicates (*n* = 3) for each condition. Box and Whisker plots show median, 25th and 75th quartile, and whiskers to 1.5 times interquartile range. **a** Polysome profile from sucrose density gradient. Unstressed control in black and stress in red. The three independent biological replicates are shown. The line “polysome” indicates the pooled fractions for MSR-seq. **b** Read coverage among RNA families for each sample. **c** Stress-induced changes in abundance of tRNA isoacceptor families on the polysome are calculated as in Fig. 3b, averaged across *n* = 3 independent biological replicates, displaying 46 isoacceptor families. **d** Normalized abundance of polysome-associated tRNA^Ile^(AAT) and tRNA^Ile^(TAT). **e** Validation of tRNA^Ile^(AAT) change on polysome by Northern blot. 5 S rRNA is the loading control. All data for *n* = 3 independent biological replicates is shown. Source data are provided as a Source Data file. **f** Abundance change for C47d-tRNA^Ser^ isodecoders in orange and T47d-tRNA^Ser^ in blue. ****p < 10^−4^, ***p < 10^−3^, ns not significant; significance calculated with two-sided *t*-test. Data were shown for *n* = 3 independent biological replicates of 8 and 6 C or T tRNA^Ser^ isodecoders, respectively. *P* values are 7.4 × 10^−4^ and 6.6 × 10^−4^ for Cand T isodecoders. **g** Stress-induced change in tRNA charging on the polysome; charging is calculated as in Fig. 3c. averaged across *n* = 3 independent biological replicates, displaying 46 isoacceptor families. **h** Comparing mutation fraction of position 32 and 47d of tRNA^Ser^ isodecoders in the total RNA (input) and on polysome of the unstressed control. C47d-tRNA^Ser^ isodecoders in orange and T47d-tRNA^Ser^ in blue. ****p < 10^−4^, *p < 0.05, ns not significant; significance calculated with two-sided *t*-test. Data were shown for *n* = 3 independent biological replicates of 8 and 6 C or T tRNA^Ser^ isodecoders, respectively. *P* values are 3.4 × 10^−8^ and 2.0 × 10^−2^ for positions 32 and 47d. **i** Stress-induced m^3^C32 and m^3^C47d change of tRNA^Ser^ isodecoders. C47d-tRNA^Ser^ isodecoders are in orange, T47d-tRNA^Ser^ in blue. Data were shown for *n* = 3 independent biological replicates of 8 and 6 C or T tRNA^Ser^ isodecoders, respectively.

We performed an MSR-seq of RNA from polysome fractions to directly reveal tRNA involvement in translational regulation during stress. The polysome profile showed a significant decrease in global translation in all three stress conditions, with the largest decrease occurring under arsenite stress (Fig. 4a). We confirmed the known increase of eIF2α phosphorylation level for arsenite stress (Supplementary Fig. 4a). We obtained on average 3.3 million mapped reads among all samples (Fig. 4b). As expected for polysome samples, the rRNA and mRNA read portions were markedly increased, and the tRNA portion decreased in the polysome compared to total RNA mapping.

The tRNA abundance change at the isoacceptor level on the polysome is the largest for the arsenite stress (Fig. 4c and Supplementary Data 3). Specifically, tRNA^Ile^(AAT), tRNA^Ile^(TAT), and tRNA^Lys^(TTT) showed a 3–4-fold increase (Fig. 4d and Supplementary Fig. 4b), while the bulk of other tRNAs showed a slight overall decrease. We validated the increase of tRNA^Ile^(AAT) under stress by Northern blot (Fig. 4e). In addition, C47d-tRNA^Ser^ isodecoders showed an increase in the polysome compared to T47d-tRNA^Ser^ in heat and arsenite stress (Fig. 4f), similar to the tRNA^Ser^ isodecoder patterns observed in the total tRNA (Fig. 3g). The overall tRNA charging level on the polysome decreased slightly in heat and increased slightly in arsenite (Fig. 4g and Supplementary Data 4). The charging levels for the bulk tRNA^Ser^ isodecoders changed little (Supplementary Fig. 4c). The most striking result of polysome tRNA abundance and charging, therefore, was the dramatic increase of tRNA^Ile^(AAT), tRNA^Ile^(TAT), and tRNA^Lys^(TTT) abundance in the arsenite stress. All three tRNAs read A/T-rich codons and may associate with ribosome stalling that contributes to the reduction of global translation (see below).

We examined how tRNA^Ser^ m^3^C modifications were associated with the polysome. In unstressed controls, polysome-associated tRNA^Ser^ showed an increase in m^3^C47d and m^3^C32 levels compared to bulk tRNA (Fig. 4h and Supplementary Fig. 4d). In heat and arsenite stress, m^3^C47d was markedly decreased on the polysome, whereas m^3^C32 levels remained the same (Fig. 4i and Supplementary Fig. 4e). The tRNA^Ser^ m^3^C response to stress can be summarized in a model of a coordinated response of m^3^C47d and m^3^C32 on the polysome. In unstressed cells, m^3^C47d and m^3^C32 levels are higher on the polysome than in the total tRNA, suggesting that m^3^C modification enhances decoding in general. In the total RNA under arsenite and heat stress, the m^3^C47d level decreases, possibly through the action of a cellular m^3^C eraser^26,27^. The m^3^C47d level also decreases on the polysome, possibly in response to the m^3^C47d level decrease in total RNA. By contrast, the m^3^C32 level increases in bulk tRNA, possibly through the action of a cellular m^3^C32 writer^28^. There is no change in m^3^C32 levels on the polysome under stress, as m^3^C32 in both C47d and T47d-tRNA^Ser^ are already at nearly stoichiometric levels as indicated by their very high mutation fractions in the total RNA (Fig. 4h).

### Stress-induced change in translation efficiency is codon usage-dependent

**Fig. 5:**
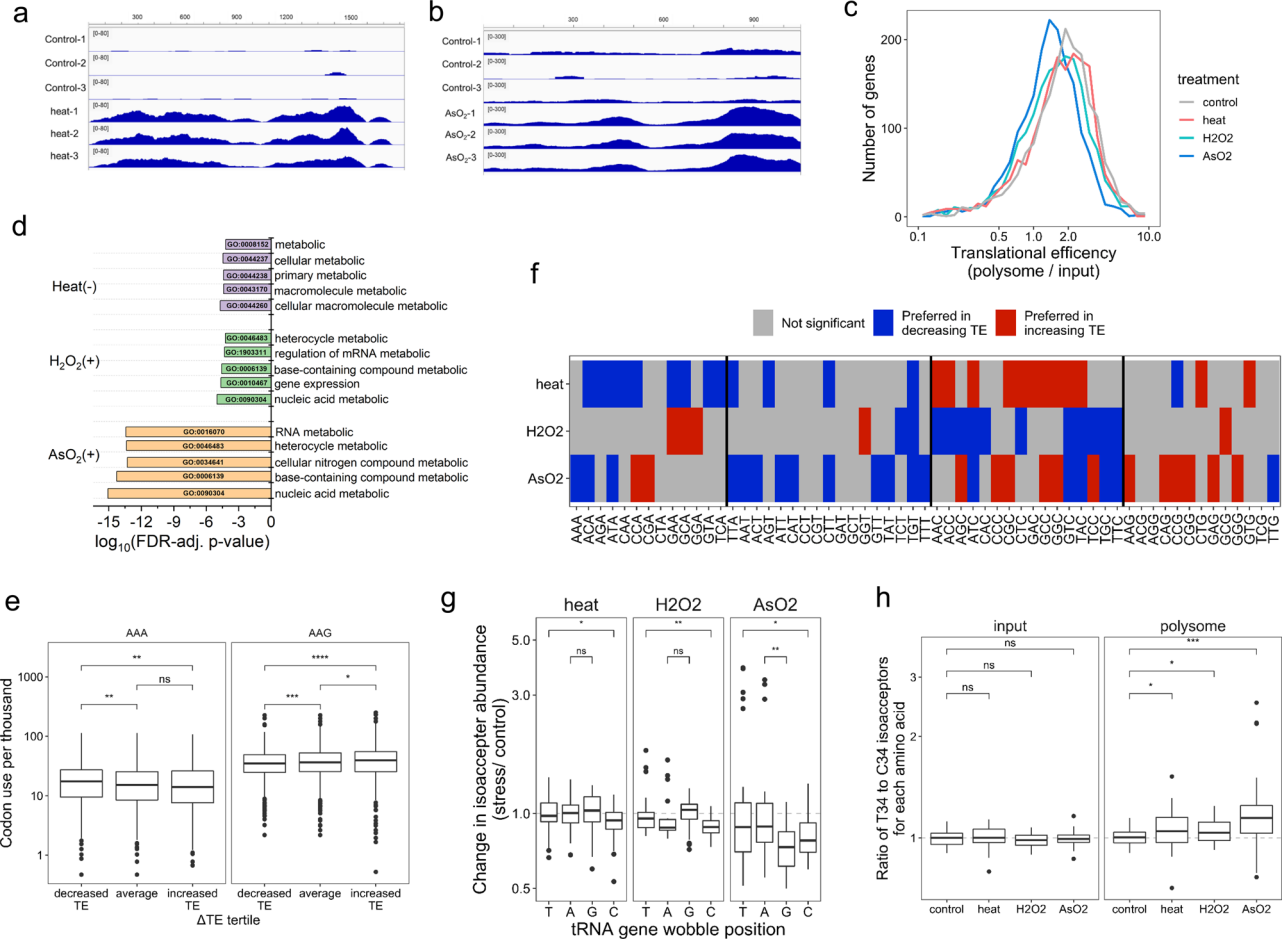
mRNA analysis of total RNA and on polysome under stress. mRNA-seq was performed in biological triplicates (*n* = 3) for each condition. Box and Whisker plots show median, 25th and 75th quartile, and whiskers to 1.5 times interquartile range. **a** IGV plot for the coding region of the HSP1A1 mRNA transcript, unstressed control (top), and heat stress (bottom) shows stress-increased read coverage along the length of the CDS. **b** IGV plot for the coding region of the ATF4 mRNA, unstressed control (top), and arsenite stress (bottom) showing stress-increased read coverage along the length of the CDS. **c** Histogram of translation efficiency (TE) among commonly detected mRNA genes in all conditions, in 30-gene increments. **d** Gene ontology analysis of biological processes among genes with stress-induced changes to TE. (+) Genes with highly increased translation efficiency, (−) Genes with highly decreased translation efficiency. **e** Lysine codon usage for AAA and AAG is compared between genes in the bottom, middle, and top tertile of ΔTE values during arsenite stress. ****p < 10^−4^, ***p < 10^−3^, **p < 0.01, *p < 0.05, ns not significant; significance calculated with two-sided Wilcoxon test. Data were shown for *n* = 3 independent biological replicates for *n* = 1747 genes, comparing tertiles. *P* values (top to bottom) for AAA are 2.6 × 10^−3^ and 9.5 × 10^−3^, and for AAG 1.7 × 10^−8^, 4.3 × 10^−2^, and 3.5 × 10^−4^. **f** Heat map showing significant differences in codon use between genes with increasing versus decreasing TE during stress. Significance is computed as Wilcox test *p* < 0.05 for comparing codon use between genes in the top and bottom tertile of ΔTE values for each stress. The heat map shows codons preferred by genes with increasing TE (red) or decreasing TE (blue) (**g**) tRNA abundance change on the polysome for isoacceptors with different wobble anticodon nucleotide in the tRNA gene. **p < 0.01, *p < 0.05, ns not significant; significance calculated with two-sided Wilcoxon test. Data were shown for *n* = 3 independent biological replicates for 42, 24, 24, and 48 isodecoders with wobble nucleotide T, A, G, or C, respectively. *P* values (top to bottom) for heat: 1.3 × 10^−2^ H_2_O_2_: 2.3 × 10^−3^ NaAsO_2_: 1.5 × 10^−2^, 5.9 × 10^−3^. **h** tRNA abundance change of the ratios for T34 versus C34 wobble anticodon tRNA for each amino acid, total RNA input on the left, and on the polysome on the right. ***p < 10^−3^, *p < 0.05, ns not significant; significance calculated with two-sided Wilcoxon test. Data were shown for *n* = 3 independent biological replicates for 11 amino acids. *P* values (top to bottom) for polysome are 7.4 × 10^−4^, 2.4 × 10^−2^, 3.7 × 10^−2^.

To gain further insights into the stress response, we sequenced polyA-selected mRNA using MSR-seq for the same total RNA and polysome profiling samples (Supplementary Fig. 5a). As expected, many mRNA transcripts changed expression under stress (Supplementary Fig. 5b). For example, heat stress increased the level of the hsp1a1 transcript by ~10-fold in total RNA, and ~30-fold in polysome-associated RNA (Fig. 5a); arsenite stress increased the eIF2α phosphorylation-dependent translation of ATF4 transcript by ~3.5-fold (Fig. 5b). We found a decrease in translation efficiency among a set of well-detected mRNAs under each stress which was particularly pronounced in arsenite stress, both globally (Fig. 5c) and for individual mRNA transcripts (Supplementary Fig. 5c). Gene ontology of the mRNAs with either highly increased or decreased translation efficiency showed the most affected genes belonging to metabolic processes (Fig. 5d).

We analyzed codon use in an effort to understand how changes in polysome-associated tRNAs may affect translation, similar to those done by Begley, Dedon, and co-workers^29–31^. We limited our analysis to a set of mRNAs that are well detected in all stress conditions and in both input and polysome fractions (~1700 genes). First, we observed that codon usage was related to transcript abundance, for example, abundant transcripts encode more lysine using both AAA and AAG codons (Supplementary Fig. 5d). Next, we compared genes with high and low translational efficiency (TE) during non-stress conditions. Genes with high TE contained significantly fewer AAA codons than genes with low TE, this pattern was absent for the synonymous codon AAG (Supplementary Fig. 5e). Expanding on this result, we asked how codon usage was related to stress-induced changes in TE. We observed that genes with decreased TE during arsenite stress contained significantly more AAA than genes with increased TE, and found the opposite pattern for the synonymous lysine codon AAG (Fig. 5e, f). We repeated this analysis for every codon in each stress to see how changes in TE were broadly related to codon usage. We found that during heat and arsenite stress, the A/T-ending codons were enriched in genes with decreasing TE, but the C/G-ending codons were enriched in the genes with increasing TE (Fig. 5f).

We then examined the relationship between changes in codon preference and translational efficiency to changes in tRNA abundance. Only A/G-ending codons are analytically tractable for this analysis since T34 wobble tRNAs can have a preference for A-ending codons and C34 wobble tRNA reads only G-ending codons. Comparing the tRNA abundance change on the polysome under stress showed a preference of T34 over C34 tRNA in all cases (Fig. 5g). The T34 over C34 tRNA preference was further confirmed by comparing the abundance change of every T34 over C34 tRNA of the same amino acid on the polysome, and the absence of this preference in total RNA under stress (Fig. 5h).

The polysome mRNA and tRNA results can be explained by a tRNA-dependent downregulation of translational elongation during stress. Even though mRNAs on the ribosome are enriched for C/G-ending codons, tRNAs on the ribosome are enriched for those that read A/T-ending codons. This is consistent with increased ribosome stalling at A/T-ending codons corresponding to slowed elongation exacerbated by stress. Potential ribosome stalling may be particularly pronounced at the Ile-AAT, Ile-ATA, and Lys-AAA codons under arsenite stress which may explain the high level of on-polysome accumulation of tRNA^Ile^ and tRNA^Lys^(TTT) that read these codons.

### tRNA modification affects tRNA fragment biogenesis

**Fig. 6:**
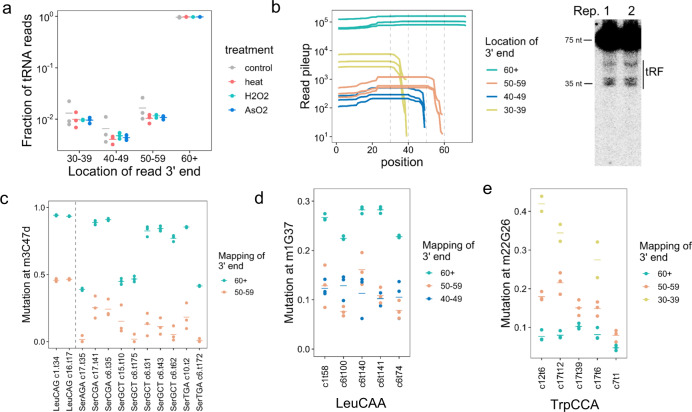
Analysis of tRNA fragment biogenesis and tRNA modification. MSR-seq was performed in biological triplicates (*n* = 3) for each condition. **a** Total count of tRNA fragment (tRF) relative to full-length tRNA in all four conditions. 60+ tRNA are considered full-length tRNAs; replicates are shown as points, mean of replicates is shown as a bar. **b** Read pileup of the most abundant tRF^Gly^(CCC) isodecoder showing the amount of tRFs with 3′ ends in 30–39 (anticodon loop), 40–49 (variable loop), and 50–59 (T loop) in unstressed control samples. The gel shows Northern blot tRF^Gly^(CCC) validation. Data for *n* = 2 independent biological replicates are shown. Source data are provided as a Source Data file. **c** Comparing mutation fraction of m^3^C47d in tRF versus full-length cognate tRNA for tRNA^Leu^(CAG) and tRNA^Ser^ isodecoders in unstressed controls. **d** Comparing mutation fraction of m^1^G37 in tRF versus full-length cognate tRNA for tRNA^Leu^(CAA) isodecoders in unstressed controls. **e** Comparing mutation fraction of m^2^_2_G26 in tRF versus full-length cognate tRNA for tRNA^Trp^ isodecoders in unstressed controls.

tRNA fragments (tRF) are a family of small RNAs that regulate many aspects of gene expression^32,33^. tRF sequencing commonly uses size-selected RNAs of 20–60 nucleotides. Although this approach obtains a high coverage of tRF and other small RNAs, potential direct connections between tRF and full-length tRNA are diminished by sequencing them separately. To simultaneously analyze tRNA and tRF in MSR-seq, we used a simplistic approach by binning reads by the 3′ ends of their mapped tRNA (Supplementary Fig. 6a). The bins roughly correspond to fragments that terminate in the T stem-loop (50–60), variable loop and adjacent region (40–50), and anticodon stem-loop (30–40). Consistent with expectations, the amount of tRF mapped in this way was ~1% of the full-length tRNA (Fig. 6a). The total amount of tRF did not change much under our stress conditions which were consistent with literature using specific stress conditions and cell lines. For example, arsenite stress showed a high level of tRF only at ≥500 µM^34,35^ whereas our stress was at 300 µM. Most studies on arsenite stress were performed with HeLa cells, whereas we used HEK293T which generated lower levels of tRF at 500 µM arsenite^36^. We validated the tRF pattern of sequencing by Northern blot (Fig. 6b and Supplementary Fig. 6b).

An important question in tRF biology is how specific tRNA modifications in the full-length tRNA affect tRF biogenesis. Reduction of modification levels by writer enzyme knock-down/knock-out has revealed that modifications such as m^5^C, Q, and m^1^G protect tRNA from cleavage^37^. However, it is unclear whether naturally occurring tRNA lacking a specific modification enhances or hinders tRF biogenesis. We addressed this question by simultaneously comparing the mutation fractions of the tRF and its cognate full-length tRNA of the same sequence. The m^3^C47d level was much lower in tRF compared to their cognate full-length tRNA (Fig. 6c and Supplementary Fig. 6c), consistent with the C47d-hypomodified tRNA being preferred substrates for tRF biogenesis. Similarly, the m^1^G37 level was lower for the tRFs compared to their cognate full-length tRNA^Leu^(CAA) (Fig. 6d and Supplementary Fig. 6d). In contrast, the m^2^_2_G26 level was higher for tRFs compared to their cognate full-length tRNA^Trp^ (Fig. 6e and Supplementary Fig. 6e). These results indicate that tRNA modification can both stimulate and inhibit tRF biogenesis. Our results also show that tRNA modification affects cleavage in the distal regions of tRNA, similar to those observed previously for m^5^C48–50 and m^1^G9 modifications^38,39^. By directly comparing the modification levels of tRF and full-length tRNAs, MSR-seq provides another avenue to investigate the mechanisms of tRF biogenesis.

## Discussion

Here we developed an RNA-seq method that enables multiplexed sequencing library preparation, on-bead enzymatic and chemical treatment, one-pot tRNA abundance, charging, and modification measurements, and simultaneous analysis of tRNA, tRNA fragment, and other small RNA families. We applied our method to human cell stress response and found additional insights into translational regulation through tRNA response to stress and in tRNA fragment biogenesis.

Advantages of carrying out sequencing library construction on solid support include the rapid exchange of buffers and reagents, thorough removal of contaminants, and elimination of size selection steps or adapter/primer removal. The solid support platform also allows for on-bead treatment of RNA with chemicals and enzymes, which has become widely used in studies of RNA modifications and other applications^40–43^. We found that the RNA libraries on streptavidin beads could withstand harsh chemical treatments such as the CMC reaction, which involves two steps at pH 8–10 and hours of incubation at 30–37 °C. Chemical and enzymatic treatments are useful in profiling RNA modifications such as Ψ, m^5^C, m^1^A, or m^7^G^44,45^ and for RNA structural mapping.

The key feature of our method is the design of the capture hairpin oligonucleotide (CHO). Our innovation is the inclusion of a 3′ ribonucleotide and 3′ phosphate which blocks the second ligation to the excess CHO upon periodate oxidation after the RT reaction so that only CHOs with cDNA product are amplified in the final PCR step. The presence of a substantial amount of the terminal transferase product (TdT)^18^ in the RT reaction is the single issue that remains to be solved. We found that the amount of TdT was highly variable depending on the input sample and reaction conditions. Currently, we remove the TdT products by size selecting the final PCR products. Possible future solutions include screening other RTs, further optimization of reaction conditions, and finding new enzymes that can selectively remove the added deoxynucleotides.

Translational regulation in stress response has been extensively studied^46,47^, and our work here reveals additional insights regarding tRNA abundance and modification. This was achieved by not only measuring the response of total tRNA, but also tRNA on the polysome under stress. Previous tRNA profiling from density gradients analyzed tRNAs in the ribo-seq 80 S peak^48^. Our tRNA profiling includes the fractions of disomes and larger which measures tRNAs only in the elongating ribosomes. Most previous stress response studies deal with the regulation of global translation and translational initiation. By measuring tRNAs on elongating ribosomes, we were able to illuminate tRNA-based stress response in translational elongation.

For tRNA abundance, the most striking result is the enrichment of tRNAs that read A-ending over G-ending codons on the polysome under stress. At the same time, mRNAs from the same polysome samples show enrichment of C/G-ending over A/T-ending codons. Among the 3 tRNAs that are enriched at the highest level on the polysome, tRNA^Ile^(AAT) reads ATT, tRNA^Ile^(TAT) reads ATA, and tRNA^Lys^(TTT) reads AAA codons. These results are consistent with increased ribosome stalling at A/T-ending codons under stress; furthermore, ATT, ATA, and AAA codons may be hotspots of such stalling under arsenite stress. Stress-induced, codon-dependent tRNA response in translational elongation may also be driven in part by the post-translational modification of the eEF2 protein^49^.

For tRNA modification, we focused on m^3^C, which can be studied by MSR-seq at high sensitivity. m^3^C32 is present in the anticodon loop of tRNA^Ser^, tRNA^Thr^, and tRNA^Arg^(yCT) and enhances translation^28^. m^3^C47d is in the variable loop of tRNA^Ser^ and tRNA^Leu^(CAG), which is located at the 40S–60S interface in the 80 S ribosome. Our striking result is the coordinated response of m^3^C32 and m^3^C47d under stress. Without stress, tRNAs with higher m^3^C32 and m^3^C47d levels are loaded on the polysome. Heat and arsenite stresses reduce the m^3^C47d level in total RNA, possibly through the action of a tRNA eraser such as ALKBH3;^26^ this results in a corresponding reduction of m^3^C47d level in polysome-associated tRNA^Ser^. However, the same stresses also increase m^3^C32 levels in tRNA^Ser^ to reach nearly stoichiometric levels in total RNA and in polysome. These results are consistent with both m^3^C modifications working together to fine-tune decoding under stress. At this time, however, the m^3^C47d installation enzyme is not known^50^, so we were unable to thoroughly investigate the function of this modification on codon-dependent protein synthesis. Once the m^3^C47d writer is identified, knock-down or knock-out of this enzyme separately or in combination with the m^3^C32 writer could reveal how these modifications coordinate in translation regulation.

We also revealed an underappreciated relationship between tRNA modification and tRNA fragment biogenesis in native tRNA. tRFs are involved in many aspects of gene expression, developmental biology, and human diseases. It is well known that tRNA modifications strongly influence tRF biogenesis, with a consensus that tRNA modification generally hinders tRF generation. We present two insights here through simultaneous full-length tRNA and tRF analysis in the same data. First, certain tRFs have lower m^3^C or m^1^G levels compared to their cognate full-length tRNA, indicating that naturally occurring hypomodified tRNAs can indeed be preferred reservoirs for tRF biogenesis. Second, tRFs can have higher m^2^_2_G levels compared to their cognate tRNA, indicating that naturally occurring tRNA modifications can also enhance tRF biogenesis.

In summary, MSR-seq provides a platform for small RNA-seq with the emphasis on RNA components in translation and translational regulation and simultaneous analysis of multiple RNA families.

## Methods

### One-pot deacylation and β-elimination for tRNA charging

Up to 500 ng of total RNA in 7 µL was used for optional one-pot beta-elimination prior to library construction. To start, 1 µL of 90 mM sodium acetate buffer, pH 4.8 was added to 7 µL input RNA. Next, 1 µL of freshly prepared 150 mM sodium periodate solution was added for a reaction condition of 16 mM NaIO_4_, 10 mM NaOAc pH 4.8. Periodate oxidation proceeded for 30 min at room temperature. Oxidation was quenched with the addition of 1 µL of 0.6 M ribose to 60 mM final concentration and incubated for 5 min. Next 5 µL of freshly prepared 100 mM sodium tetraborate, pH 9.5 was added for a final concentration of 33 mM. This mixture was incubated for 30 min at 45 °C. To stop β-elimination and perform 3′ end-repair, 5 µL of T4 PNK mix (200 mM Tris-HCl pH 6.8, 40 mM MgCl_2_, 4 U/µL T4 PNK, from New England Biolabs) was added to the reaction and incubated at 37 °C for 20 min. T4 PNK was heat-inactivated by incubating at 65 °C for 10 min. This 20 µL reaction mixture can be used directly in the first barcode ligation by adding 30 µL of a ligation master mix described below.

### Standard tRNA deacylation

Total RNA was prepared for library construction by the first deacylating in a solution of 100 mM Tris-HCl, pH 9.0 at 37 °C for 30 min, then neutralized by addition of sodium acetate, pH 4.8 to a final concentration of 180 mM. Deacylated RNA was then ethanol precipitated and resuspended in water, or desalted using a Zymo Research Oligo Clean-and-Concentrator spin column.

### General protocol for MSR-seq

#### First barcode ligation

Depending on the nature of the experiment described in the main text, input RNA or total nucleic acid samples were either deacylated or had undergone one-pot deacylation and β-elimination as described above. Up to 1 µg of total RNA input was used in a ligation reaction of 50 µL with the following components: 1 U/µL T4 RNA ligase I (NEB), 1x NEB T4 RNA ligase I buffer, 15% PEG 8000, 50 µM ATP, 1 mM hexamine cobalt chloride, and 5% DMSO. After adding the ligation mix to the sample, the capture hairpin oligo (CHO, Supplementary Data 5) was added to a final concentration of 1 µM and the samples were incubated at 16 °C overnight (12+ h).

#### Binding to dynabeads

The ligation mixture was diluted by adding an equal volume of water to reduce the viscosity of the solution. Next, streptavidin-coated MyOne C1 Dynabeads (Thermo Fisher) were added to each sample in a 1.2:1 excess over CHO (for example, a 50 µL reaction had 50 pmol capture hairpin oligo; beads were supplied at 10 mg/ml and had a binding capacity of 500 pmol biotinylated oligo per mg, so use 12 µL beads). The bead-sample mixture was incubated at room temperature for 15 min. After binding, supernatants were removed, and the beads were washed once with high salt wash buffer (1 M NaCl, 20 mM Tris-HCl, pH 7.4) and once with low salt wash buffer (100 mM NaCl, 20 mM Tris-HCl, pH 7.4). After washing, multiple individually barcoded samples can be combined for downstream steps. At this stage, enzymatic or chemical treatments can be incorporated into the library preparation protocol such as AlkB demethylase reaction or CMC treatments (see below).

#### Dephosphorylation

A 50 µL dephosphorylation mix containing the following was added to the multiplexed sample on bead: 0.04 U/µL calf-intestine phosphatase (Roche), 10 mM MgCl_2_, 0.5 mM ZnCl_2_, 20 mM HEPES, pH 7.3. The sample was incubated at 37 °C for 30 min. The sample was then washed once with high salt wash buffer and once with low salt wash buffer, then resuspended in 20 µL water.

#### Reverse transcription

Five microliters of SuperScript IV VILO 5x master mix (Thermo Fisher) were added to the dephosphorylated sample (final volume of 25 µL) and then incubated at 55 °C for 10 min. The sample was then washed once with high salt wash buffer and once with low salt wash buffer. Identical results were obtained upon extending the reaction time to 60 min. For overnight reverse transcription, after the initial 10 min incubation at 55 °C, the samples were further incubated at 35 °C overnight.

#### RNase H digestion

Beads were then resuspended into 50 µL of RNase H master mix containing 0.4 U/uL RNase H (NEB) and 1x NEB RNase H buffer and incubated at 37 °C for 15 min. The sample was then washed once with high salt wash buffer and once with low salt wash buffer. The sample was then resuspended in 40 µL water.

#### Periodate oxidation

Ten microliters of 250 mM freshly prepared sodium periodate, 0.5 M sodium acetate, pH 5 were added to the RNase H digested sample and incubated at room temperature for 30 min. Afterward, ribose was added to a final concentration of 167 mM to quench excess periodate at room temperature for 5 min. The sample was then washed once with high salt wash buffer and once with low salt wash buffer.

#### Second ligation

Beads were resuspended into 50 µL of a ligation master mix with the following components: 2 U/µL T4 RNA ligase I (NEB), 1x NEB T4 RNA ligase I buffer, 2 µM second ligation oligo (Supplementary Data 5), 25% PEG 8000, 50 µM ATP, 7.5% DMSO, and 1 mM hexaammine cobalt chloride. After incubation at room temperature overnight (12+ h), the reaction was diluted with 50 µL water to reduce viscosity, washed once with high salt wash buffer and once with low salt wash buffer, and then resuspended in water. The amount of water was 6 µL per sample in the first ligation reaction, before pooling the barcoded samples. For example, if the second ligation mixture contains a pool of six samples, the amount of water used for resuspension would be 36 µL. Samples can be stored at 4 °C or frozen at −20 °C; both can be used for the next PCR step.

#### PCR

A 50 µL PCR reaction was run using 5–10% of the bead slurry products from the second ligation reaction using Q5 DNA polymerase (NEB) and following the manufacturer’s instructions: 0.02 U/µL Q5 DNA polymerase, 1x Q5 reaction buffer, 0.2 mM dNTPs, 0.5 µM Illumina index primer (Supplementary Data 5), and 0.5 µM Illumina multiplex primer (Supplementary Data 5). Typical PCR cycles were 9–15 cycles at 10 s at 98 °C, 15 s at 55 °C, and 72 °C for 20 s. PCR reactions were then processed through the DNA Clean and Concentrate kit (Zymo Research).

#### TBE-PAGE gel extraction

Following desalting, PCR products were run on 10% non-denaturing TBE gel with dsDNA size markers; lanes were cut according to the desired product size, mashed by pipette tip, and then resuspended in crush-and-soak buffer (500 mM sodium acetate, pH 5.0). The gel fragments were extracted overnight and then ethanol precipitated.

### First ligation bias test

Four separate mixtures containing final 0.8 µM CHO and four 11-mer RNA oligonucleotides (5′ N_10_X, where X = rA, rC, rG, rU) were made at an approximate molar ratio of 2 CHO: 1 RNA oligo. Four microliters of each mixture were added to individual tubes (*n* = 3 for each RNA oligo). To each tube, 21 µL of first ligation mixture (18% PEG 8000, 1.2x NEB T4 RNA ligase I buffer, 60 µM ATP, 6% DMSO, 1.2 mM hexaammine cobalt (III) chloride, and 2 U/µL NEB T4 RNA ligase I) were added (final volume of 25 µL) and the samples were incubated at 16 °C overnight.

After overnight incubation, 2.5 µL of 18 mg/mL Roche PCR-grade Proteinase K and 15 µL of 2x RNA loading dye (9 M urea, 0.02% bromophenol blue, and 0.02% xylene cyanol) were added to each sample to degrade the ligase. The samples were then incubated at 37 °C for 25 min and subsequently boiled at 92 °C for 3 min.

The samples were loaded onto a Bio-Rad pre-cast 15% TBE-Urea PAGE gel and electrophoresed until the bromophenol blue was near the bottom of the gel. The gel was then stained with a 10,000x dilution of SYBR Gold and imaged on a Chemi-Doc imager and quantified with Quantity One software (Bio-Rad).

### Second ligation bias test

Four separate mixtures containing the final 2 µM second ligation oligo and four 26-mer 5′ biotinylated DNA oligonucleotides (5′ biotin- CTCTTCCGATCTAGT N_10_X, where X = dA, dC, dG, T) were made at an approximate molar ratio of 10 s ligation oligo: 1 DNA oligo. Four microliters of each mixture were added to individual tubes (*n* = 3 for each biotin-DNA oligo). Three microliters of 10 mg/mL Thermo Fisher streptavidin-coated MyOne C1 Dynabeads were then added to each sample and allowed to capture the biotinylated DNA oligos for 10 min.

Then, to each tube, 21 µL of second ligation mixture (30% PEG 8000, 1.2x NEB T4 RNA ligase I buffer, 60 µM ATP, 9% DMSO, 1.2 mM hexaammine cobalt (III) chloride, and 4 U/µL NEB T4 RNA ligase I) was added (final volume of 25 µL) and the samples were incubated at room temperature (~20 °C) overnight.

After overnight incubation, 2.5 µL of 18 mg/mL Roche PCR-grade Proteinase K and 15 µL of 2x RNA loading dye (9 M urea, 0.02% bromophenol blue, and 0.02% xylene cyanol) were added to each sample to release the products from the beads and to degrade the ligase. The samples were then incubated at 37 °C for 25 min and subsequently boiled at 92 °C for 3 min.

The samples were loaded onto a Bio-Rad pre-cast 15% TBE-Urea PAGE gel and electrophoresed until the bromophenol blue was near the bottom of the gel. The gel was then stained with a 10,000x dilution of SYBR Gold and imaged on a Chemi-Doc imager quantified with Quantity One software (Bio-Rad).

### On-bead RT and phosphatase (CIP) treatment validation

A first ligation mixture was made containing the following: 15% PEG 8000, 1x NEB T4 RNA ligase I buffer, 50 µM ATP, 1 mM hexaammine cobalt (III) chloride, 0.8 µM CHO, 25 ng/µL HEK total RNA, and 1.67 U/µL NEB T4 RNA ligase I (total volume of 275 µL). This was incubated at 16 °C overnight.

After overnight ligation, 75 µL (equivalent to 3 × 25 µL ligation reactions) were pipetted into a separate tube (henceforth known as “- CIP” tube) and mixed with 75 µL of 100 mM EDTA to quench the ligation reaction. The remaining ligation mixture was ethanol precipitated.

The ethanol-precipitated ligation mixture (henceforth known as “+CIP” tube) was resuspended in 92 µL of water and mixed with 100 µL of 2x dephosphorylation buffer (20 mM MgCl_2_, 1 mM ZnCl_2_, and 40 mM HEPES, pH 7.5) and 8 µL of 5 U/µL NEB Quick CIP. The “+ CIP” tube was then incubated at 37 °C for 30 min. After the 37 °C incubation, the “+CIP” tube was incubated at 65 °C for 10 min to inactivate the phosphatase. The “+ CIP” sample was then ethanol precipitated.

After ethanol precipitation, the material in the “+ CIP” tube was resuspended in 64 µL of water. Forty-eight microliters were then pipetted out and split into six separate tubes (8 µL each). To three of these tubes, 8 µL of 10 mg/mL Thermo Fisher streptavidin-coated MyOne C1 Dynabeads were added. To the “−CIP” tube, 24 µL of beads were added. The beads were incubated with the samples for 10 min to allow them to bind the biotinylated oligos. (The “−CIP” tube was then split into three separate tubes.) After incubation, the beads were then magnetized, the supernatant was removed, and the beads were washed once with 50 µL of high salt Neonate wash buffer (20 mM Tris, 1 M NaCl, and 0.1% Neonate 20, pH 7.4) and then washed once with low salt wash buffer (20 mM Tris and 0.1 M NaCl, pH 7.4).

After washing, the samples were resuspended in 8 µL of autoclaved water. To all nine tubes, 2 µL of Thermo Fisher 5x SuperScript IV VILO mix were added and the samples were then incubated at 55 °C for 10 min. After incubation, the off-bead samples were then mixed with 8 µL of beads. All samples were then washed as before.

From this point on, the MSR-seq protocol was followed, beginning at the *RNase H digestion* section.

### Oligonucleotide sequences

See Supplementary Data 5.

### m^3^C poisoned primer extension

About 100 ng of total RNA sample was added to a tube containing 20 pmol (2 μM final concentration) of RT primer and 2 μL of 5x annealing buffer (10 mM KCl and 150 mM Tris-HCl, pH 7.5); water was added for a total volume of 5 μL. Samples were heated to 93 °C for 2 min and then directly placed on ice. Five microliters of a Post-annealing mix, containing the following components, was added to each sample tube (10 μL final volume): 2x AMV Buffer; 2 U/μL AMV RT [New England Biolabs]; 2x poisoned dHTP mix (2 mM dATP, 2 mM dCTP, and 4 mM ddTTP); and 2 μCi/μL α-^32^P dGTP [Perkin Elmer]. The samples were incubated at 37 °C for 30 min; and then mixed with 10 μL of 2x urea loading dye (9 M urea, 2 mM EDTA, 0.2% xylene cyanol, 0.2 % bromophenol blue) and incubated at 93 °C for 2 min before loading on a 15% denaturing polyacrylamide gels. The primer sequence for tRNA^Ser^(GCT) was 5′-TGGCGACGAGGATGGGATTCGAACCCACGCGT.

### AlkB and AlkB-D135S purification

These were adapted from the previously described protocols for DM-tRNA-seq^5^. Briefly, NEB T7 Expression cells were grown in LB media at 37 °C, in the presence of 50 µM kanamycin, to an A_600_ of 0.6–0.8. Once the cells reached the desired density, IPTG and iron sulfate were added to final concentrations of 1 mM and 5 µM, respectively. After induction, the cells were incubated overnight at 30 °C. Cells were collected, pelleted and then resuspended in lysis buffer (10 mM Tris, pH 7.4, 5% glycerol, 2 mM CaCl_2_, 10 mM MgCl_2_, 10 mM 2-mercaptoethanol) plus 300 mM NaCl. The cells were lysed by sonication and then centrifuged at 17,400 rcf for 20 min. The soluble proteins were first purified using a Ni-NTA superflow cartridge (Qiagen) with buffers A (lysis buffer plus 1 M NaCl for washing) and B (lysis buffer plus 1 M NaCl and 500 mM imidazole for elution) and then further purified by ion-exchange (Mono S GL, GE Healthcare) with buffers A (lysis buffer plus 100 mM NaCl) for column loading and B (lysis buffer plus 1.5 M NaCl) for elution.

### AlkB treatment

Demethylase buffer conditions were modified from Li et al.^51^. Three stock solutions are made fresh immediately before reaction: l-ascorbic acid 200 mM, 2-ketoglutarate 3 mM, and ammonium iron sulfate 5 mM. The final reaction mixture contained 2 mM l-ascorbic acid, 1 mM 2-ketoglutarate, 0.3 mM ammonium iron sulfate, 100 mM KCl, 50 mM MES pH 6, 50 ng/µL BSA, 4 µM wild-type AlkB, and 4 µM AlkB-D135S. About 50 µL of the reaction mixture was added to 5–20 µL of decanted streptavidin bead slurry after ligation, immobilization, and washing. The reaction continued for 30 min at 37 °C. Following the reaction, beads were washed once with high salt wash buffer (1 M NaCl, 20 mM Tris-HCl pH 7.4) and once with low salt wash buffer (100 mM NaCl, 20 mM Tris-HCl pH 7.4).

### HEK cell culture and RNA extraction

HEK293T cells (ATCC, CRL-11268) were cultured with complete DMEM medium under standard conditions according to ATCC. Briefly, HEK293T cells were grown in Hyclone DMEM medium (GE Healthcare Life Sciences, SH30022.01) with 10% FBS and 1% Pen–Strep (Penicillin–Streptomycin) to 80% confluency and passaged. Cells were collected and total RNA was extracted using TRIzol (Thermo Fisher, 15596026) by following the manufacturer’s protocol when cells reached 80–90% confluency.

### Stress treatments

HEK293T cells were cultured in DMEM medium (GE Healthcare Life Sciences, SH30022.01) with 10% FBS and 1% Pen–Strep (Penicillin–Streptomycin). Twenty-four 15-cm plates of HEK293T cells (5 × 10^6^ cells each) were seeded three days before collection. On the day of polysome profiling, six plates of cells (two plates for one sample) were treated with different stress conditions: (1) unstressed control; (2) 42 °C heat shock, 1 h; (3) 0.6 mM H_2_O_2_, 2 h; (4) 300 µM NaAsO_2_, 2 h. Polysome profiling was immediately performed after stress treatment.

### Polysome profiling

Polysome profiling procedures were adapted from ref. ^52^. Briefly, cells were treated with 100 µg/ml cycloheximide (CHX) in DMEM for 7 min right after stress treatments. DMEM medium was removed. Cells were then collected using 10 ml ice-cold PBS with 100 µg/ml CHX and cell lifter. Cells were pelleted by centrifugation at 3000 RPM for 5 min. The cell pellet was washed twice with 5 ml ice-cold PBS with 100 µg/ml CHX. Cells from two plates were combined for one sample. Cells were resuspended in 1 ml ice-cold PBS and transferred to microcentrifuge tubes. About 200 µl cell suspension from each sample was saved as input. Cells were pelleted by centrifugation at 3000 RPM for 5 min. For input cells samples, 500 µl TRIzol reagent was added to extract the total RNA. For polysome cells samples, the cell pellet was resuspended by 4 volumes of lysis buffer (20 mM HEPES, pH 7.6, 100 mM KCl, 5 mM MgCl_2_, 1% Triton X-100, 100 µg/ml CHX, freshly added 1× protease inhibitor (11873580001, Roche), 40 U/µl RNase inhibitor (AM2696, Thermo)). Cells were rotated and lysed at 4 °C cold room for 30 min. Lysed samples were centrifuged at 16,000×*g* for 15 min to collect the clear lysate (~600–700 µl). About 4 ul Turbo DNase was added to each lysate and the samples were incubated at room temperature for 15 min. The samples were centrifuged again at 16,000×*g* for 15 min to get clear lysate. Absorbance at 260 nm of each sample was measured. Samples were adjusted to the same absorbance using lysis buffer. 5–50% sucrose gradient (20 mM HEPES, pH 7.6, 100 mM KCl, 5 mM MgCl_2_, 100 µg/ml CHX, freshly added 1 × protease inhibitor, 40 U/µl RNase inhibitor) was prepared using a Biocomp gradient station. About 600 µl gradient buffer was removed from the top of the balanced sucrose gradient. About 600 µl lysate was loaded onto the top of the gradient slowly while gently rotating the tube. The samples were centrifuged at 28,000 RPM for 3 h at 4 °C using a Beckman SW28.1 rotor. After centrifugation, fractions were collected and measured using the Biocomp gradient station (30 fractions total). Fractions were flash-frozen and stored at –80 °C before RNA extraction and library construction. For polysome RNA extraction, fractions from disome and higher were combined and 2 volumes of TRIzol reagent were added to extract the RNA. PolyA+ RNA was extracted from the input and polysome RNA using a polyA+ RNA extraction kit from Promega (Z5310) or NEB (E7490S), respectively.

Poly(A)-selection was done with NEBNext Poly(A) mRNA Magnetic Isolation Module (NEB, E7490S) according to the manufacturer’s instructions.

One microgram of input polyA+ RNA samples and 100 ng polysome polyA+ RNA samples were used to build sequencing libraries. The RNA fragmentation and end-repair steps are the same as the CMC sequencing libraries construction. Briefly, polyA+ RNA in 18 µl were added to PCR tubes and 2 µl Magnesium RNA fragmentation buffer (NEB, E6150S) was added to each tube. The tubes were incubated at 94 °C in a thermocycler for 5 min to fragment the RNA to ~200 nt. The tubes were transferred to ice and a 2 µl RNA fragmentation stop solution was then added to each tube to stop the fragmentation. Samples were spun down and diluted to 50 µl using sterile H_2_O. The fragmented RNA was purified using Zymo RNA clean and concentrator columns and eluted in 16 µl sterile H_2_O. Two microliters of T4 PNK buffer and 2 µl T4 PNK were added to the tubes and the tubes were incubated at 37 °C for 30 min to repair the ends of the fragmented RNA. MSR-seq method was then used to build sequencing libraries with the fragmented RNA. Slight modifications were made to the MSR-Seq protocol. After the first ligation step, the ligation reaction was quenched by adding 50 mM EDTA. Samples were combined and ligation products over 200 nt long was purified using Zymo RNA clean and concentrator columns twice. Target cDNA products were purified using AMPure XP beads with a 1:1 ratio after PCR.

### mRNA transcriptome mapping

Raw 100 bp paired-end sequencing reads were obtained from the Illumina Nova-Seq platform. Reads processing and trimming was the same as CMC sequencing libraries. The reads were mapped to the human transcriptome (hg38) obtained from Ensembl. The mapped “bam” files were then analyzed using bamCoverage tool of the “deeptools”^53^ (https://deeptools.readthedocs.io/en/develop/) with bin size as 1 to get the bigwig sequencing depth coverage files. The bigwig coverage files were visualized using IGV and coverage track images were obtained.

### Northern blots

The northern blot method was adapted from ref. ^54^ using ^32^P radiolabeled probes. About 500 ng of each polysome RNA sample were diluted to 9 µl in microcentrifuge tubes. About 1 µl 1 M Tris-HCl, pH 9 was added to each tube and mixed well. The samples were incubated at 37 °C for 30 min to deacylate the tRNAs. About 10 µl 2× RNA loading buffer ((9 M Urea, 100 mM EDTA, pH 8, 0.2% Bromophenol blue, 0.2% Xylene cyanol)) were added. All RNA samples were loaded onto a 10% pre-run denaturing PAGE gel. The gel was stopped when the Xylene cyanol band passed the middle of the gel. RNA was transferred to Hybond-XL Membrane (RPN303S, GE Healthcare) at 80 °C for 4 h using a gel dryer (Bio-Rad). The membrane was soaked in deionized water with the membrane side on the top to separate the gel and the membrane. The gel was stained with SYBR gold (S11494, Thermo) and scanned using a Chemi-Doc imaging system (Bio-Rad). The membrane was UV-crosslinked twice (254 nm for 1200 mJ). The membrane was then prehybridized for 30 min twice with hybridization buffer (20 mM phosphate, pH 7, 300 mM NaCl, 1% SDS). About 40 pmol of the tRNA probes were radiolabeled by T4 PNK with γ-^32^P-ATP in a 10 µl reaction. The labeling mixture were diluted to 50 µl and cleaned by Illustra MicroSpin G-25 Columns (27532501, Cytiva). The membrane was incubated with 15 µl ^32^P radiolabeled probes for 16 h at 60 °C in the UVP Hybridizer Oven (95-0030-01, Analytik Jena). The membrane was washed twice using 50 ml washing buffer (20 mM phosphate, pH 7, 300 mM NaCl, 2 mM EDTA, and 0.1% SDS) for 30 min each. The membrane was wrapped in plastic wrap and exposed to a phosphorimager screen for 1–2 days depending on the signal strength. The screen was then scanned using a personal molecular imager (Bio-Rad). The image was analyzed using ImageLab software.

Northern blot probes were from Integrated DNA Technologies (IDT) and gel purified. Sequences of the probes were (Y = C/T; R = A/G; W = A/T, M = A/C):

Gly: 5′-TGCATTGGCCRGGAATYGAACCCGGGYCTCCCRCGTGGWAGGCGAGAATTCTACCACTGMACCACCMAYGC-3′

Ile-AAT: 5′-TGGCCMGTACGGGGATCGAACCCGCGACCTTGGCGTTATTAGCACCACGCTCTAACCAACTGAGCTAACCRGCC-3′

### Gene ontology analysis

Gene ontology analysis was performed using the default setting in ref. ^55^ (http://geneontology.org/).

### Western blots of eIF2α phosphorylation

All samples were incubated at 95 °C for 10 min, separated on a 4–12% polyacrylamide Bis-Tris protein gel (NP0322BOX, Thermo), and transferred to polyvinylidene fluoride membranes (IPVH00010, Millipore). The membranes were blocked in 10% w/v milk (1706404, Bio-Rad). The blots were probed with 1/1000 v/v EIF2S1 antibody (AHO0802, Invitrogen) or 1/500 v/v Phospho-EIF2S1 (Ser51) antibody (MA5-15133, Invitrogen), followed by 1/10000 v/v sheep anti-mouse IgG (NA931V, Cytiva) or 1/10000 v/v donkey anti-rabbit IgG conjugated to horseradish peroxidase (NA934V, Cytiva). The blots were visualized with ECL Prime Western Blotting Detection Reagents (RPN2232, Amersham) using a Bio-Rad Chemi-Doc MP.

### MCF7 growth and RNA extraction

MCF7 cells (ATCC, HTB-22) were cultured in EMEM medium (ATCC, 30-2003) with 10% FBS (Thermo Fisher, 10082147), 0.01 mg/ml bovine insulin (Sigma-Aldrich, I0516), and 10 nM β-estradiol (Sigma- Aldrich, E2758) to 80% confluency and passaged at ratios of 1:3. Total RNA were extracted using TRIzol.

### CMC treatment/library construction

MCF7 total RNA sequencing libraries were constructed as follows. Small RNA (<200 nt) was first removed from 1 μg MCF7 total RNA using spin columns (Zymo Research RNA Clean & Concentrator-5, R1016) and the large RNA (>200 nt) was eluted with 18 μl sterile H_2_O in a microcentrifuge tube. The RNA was transferred to PCR tubes and 2 μl Magnesium RNA fragmentation buffer (NEB, E6150S) was added to each tube and the tubes were incubated at 94 °C in a thermocycler for 5 min to fragment the RNA to ~200 nt. Two microliters of RNA fragmentation stop solution were then added to each tube. The samples were diluted to 50 μl with H_2_O and Zymo Research spin columns were used to purify the fragmented RNA; the RNA were eluted in 16 μl sterile H_2_O in a microcentrifuge tube. For 3′ end-repair of the RNA fragments, 2 μl 10x T4 PNK buffer and 2 μl T4 PNK at 10 U/μl (Thermo Fisher, EK0032) were added and the mixture was incubated at 37 °C for 30 min. The fragmented, end-repaired RNA was used to build sequencing libraries using the msRNA-seq protocol described above with the following modifications. The fragmented RNA was ligated to barcoded capture hairpin oligonucleotides and bound to streptavidin beads. The samples were then pooled, mixed, and split into two parts for ±CMC (N-cyclohexyl-N′-(2-morpholinoethyl)carbodiimide) treatment (+CMC:-CMC = 1.5: 1 ratio). About 12 μl sterile H_2_O and 24 µl TEU buffer (50 mM Tris-HCl (pH 8.3), 4 mM EDTA, 7 M urea) were first added to each tube, then 4 µl freshly prepared 1 M CMC in TEU buffer was added to +CMC samples and 4 µl sterile H_2_O was added -CMC samples. The samples were incubated at 30 °C for 16 h at 1400 rpm on an Eppendorf ThermoMixer. The samples were washed twice with high salt buffer and once with low salt buffer. The samples were then resuspended with 40 µl of 50 mM sodium carbonate and 2 mM EDTA (pH 10.4) buffer and incubated at 37 °C for 6 h at 1400 rpm. The beads were washed twice with high salt buffer and once with low salt buffer and then proceeded to the msRNA-seq steps such as phosphatase treatment and reverse transcription.

### Read processing and mapping

Libraries were sequenced on Illumina Hi-Seq or NEXT-seq platforms. First, paired-end reads were split by barcode sequence using Je demultiplex with options BPOS = BOTH BM = READ_1 LEN = 4:6 FORCE = true C = false^56^. BM and LEN options were adjusted for samples with a 3 nt barcode instead of 4, and for samples where the barcode is located in read 2. Barcode sequences are listed in Supplementary Data 5. Next, only the read beginning with the barcode (usually read 2) was used to map with bowtie2 (version 2.3.3.1) with the following parameters: “-q -p 10 -local -no-unal”. For human sample reads were mapped to the human transcriptome, with tRNA genes shaped for a curated, non-redundant, set of high-scoring tRNA genes. This reference was a combination of HG19 ORFs, ncRNAs, and our curated tRNA list based on HG19 tRNAs curated to be non-redundant, tRNA-scan SE with score >47, and 3′ “CCA” appended. Bowtie2 output sam files were converted to bam files, then sorted using samtools. Next IGV was used to collapse reads into 1 nt window. IGV output.wig files were reformatted using custom python scripts (available on GitHub). The bowtie2 output Sam files were also used as input for a custom python script using PySam, a python wrapper for SAMTools (ref. ^57^, https://github.com/pysam-developers/pysam) to sum all reads that mapped to each gene. Related custom scripts were used to divide reads based on which 10 nt window the 3′ end mapped to for each tRNA; this is for fragment analysis. Data were visualized with custom R scripts. All custom scripts are available on GitHub (https://github.com/ckatanski/CHRIS-seq).

### Translational efficiency and mRNA codon usage analysis

For each gene, read counts mapping to all transcript variants were summed together. Read counts were normalized for total detection in each sample (i.e., reads per million) among mRNA-detection reads (only genes with “gene_biotype” as “protein_coding” were included for normalization). After normalization, genes were filtered to have more than 100 counts. Next translational efficiency (TE) was calculated as the normalized gene counts in the polysome fraction divided by the input fraction for each replicate and each stress. Next, analysis was limited to a set of well-detected genes: this set of genes was defined as genes where TE could be calculated in all four treatments (control, heat, H_2_O_2_, and AsO_2_) for one replicate. This gave a set of ~1500 genes, with small fluctuations in detection in other replicates. TE calculations were confirmed to be roughly log-normally distributed. A *Z*-score was calculated for each sample based on log10 (TE) value. The mean *Z*-score from the control replicates was used as a reference for stress-dependent change.

For each CDS transcript in our HG19-derived reference genome, the occurrence of each codon was tallied—frequency was calculated as the number of codon instances divided by protein length. For genes with several transcripts, the median value for each codon was used. The calculation was done with a custom python script, available on GitHub. These data were combined with our TE calculations.

Next, genes were divided into three groups: low TE, average TE, and high TE. Divisions were based on the 33rd percentile and 66th percentile rank for TE in each sample. For each codon, the frequency was used to assign a percentile rank to each gene in each sample (e.g., a gene with abundant “AAA” Lys usage may be in the 99th percentile for “AAA” and the 5th percentile for “CCT” Pro). Percentile ranks were used to calculate statistical differences between gene groups for each sample and each codon; tests were calculated via the two-sided Wilcox test. Codon usage frequency is not normally distributed, so a non-parametric hypothesis test is appropriate.

Next, for each sample, genes were again divided into three groups, but based on the percentile of change in *Z*-score from the mean of control replicates: decreased TE, no change in TE, and increased TE. Percentile group thresholds were again 33rd and 66th. Percentile ranking in codon usage was again used for hypothesis testing between groups for each sample and each codon with a two-sided Wilcox test as above.

### Read processing from CMC reaction

Raw 100 bp paired-end sequencing reads were obtained from the Illumina Hi-Seq platform. Read1 reads were separated by barcodes with the barcodes sequence on paired read2 reads using custom python scripts. Read2 reads were separated by barcodes using fastx_barcode_splitter (fastx_toolkit, http://hannonlab.cshl.edu/fastx_toolkit/). For read1 reads, the random six nucleotides' unique molecular identifier (UMI) sequence at the start of the reads and the barcoded adapter sequence at the end of the reads were removed using Trimmomatic^58^ using single-end mode with a 15 nt cutoff. For read2 reads, the 7 nt barcode sequence at the start of the reads and the UMI and adapter sequence at the end of the reads were removed by Trimmomatic using paired-end mode with a 15 nt cutoff. The reads were then mapped to human rRNA transcripts using bowtie2. The output sam files were converted to bam files and then sorted and indexed using samtools. A command-line version of “igvtools count” (IGV, http://software.broadinstitute.org/software/igv/download) were used to count nucleotide composition, insertions, and deletions at single-base resolution. “Bedtools genomecov” (bedtools, https://bedtools.readthedocs.io/en/latest/) was used to count the start and end of all reads at each position. All the output files and reference sequence were combined into a single file for each sample, and the mutation rate and the stop rate were computed by custom python scripts. The output files were analyzed to identify target pseudouridine sites.

### Reporting summary

Further information on research design is available in the Nature Research Reporting Summary linked to this article.

## Supplementary information

Supplementary Information

Peer Review File

Description of Additional Supplementary Files

Supplementary Data 1

Supplementary Data 2

Supplementary Data 3

Supplementary Data 4

Supplementary Data 5

Reporting Summary

## Data Availability

The data supporting the findings of this study are available from the corresponding authors upon reasonable request. The sequencing data generated in this study have been deposited in the NCBI Geo database under accession # GSE198441. The processed sequencing data are available at GSE198441. These data were associated with Figs. 2–6 and Supplementary Figs. 2–6.  Source data are provided with this paper.

## Source data

Source Data

## Code availability

Custom scripts for read processing are available at https://github.com/ckatanski/CHRIS-seq. Scripts for data processing and figure creation are also available at https://github.com/ckatanski/CHRIS-seq.
