# Supplementary material for: A multiplex platform for small RNA sequencing elucidates multifaceted tRNA stress response and translational regulation
Source: Nat Commun. 2022 May 5;13:2491. doi: 10.1038/s41467-022-30261-3 (PMC9072684; doi:10.1038/s41467-022-30261-3)
Supplement: Supplementary file 1 — Supplementary Information [file 41467_2022_30261_MOESM1_ESM.pdf]

Supplementary Information for

A multiplex platform for small RNA sequencing elucidates multifaceted tRNA stress response and translational regulation

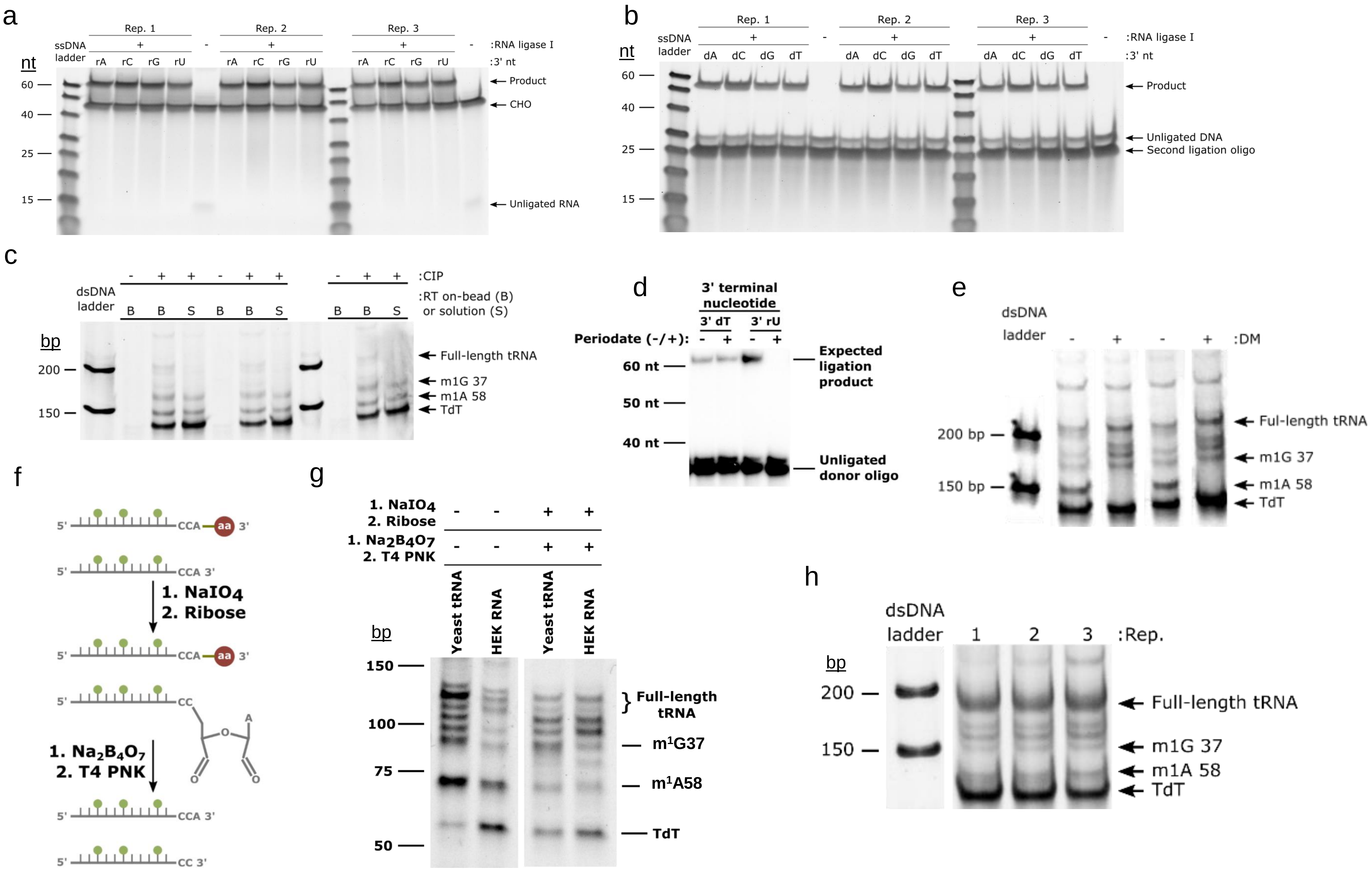

Supplementary Figure 1: legend on next page.

Supplementary Figure 1: Optimization of MSR-seq.

Source data are provided as a Source Data file for 1a, 1b, 1c, 1d, 1e, 1g, 1h.

(a) First ligation of synthetic ribo-oligonucleotides to capture hairpin in solution. Samples inputs were four RNA 11mers of 5’ N_{10}-A/C/G/U (labels indicate the identity of the 3’ nucleotide of the substrate RNA i.e. ribo-uricil = rU). Input RNAs were ligated to capture hairpin oligo (CHO) and ligation products were separated on 15% TBE-urea gel and stained with SYBR Gold. Results are quantified in Figure 1c. All data from n=3 biological replicates is shown.

(b) Second ligation of synthetic deoxy-oligonucleotides to ligation oligo donor on streptavidin beads. Sample inputs were four 5’ biotinylated DNA 26mers ending with dN_{10}-dA/dC/dG/T intended as mock cDNA products featuring different terminal nucleotides. The ligation oligo donor was the same used for ligation to the cDNA product on bead in the library construction. Ligation reaction products were separated on 15% TBE-Urea gel and stained with SYBR Gold. Results are quantified in Figure 1d. All data from n=3 biological replicates is shown.

For panels (c), (e), (g), (h), DNA size markers are on the left. Major RT stops caused by the base methylations (m^1A58, m^1G37) in human tRNAs are indicated on the right. TdT corresponds to the product derived from the aberrant terminal transferase activity of the RT.

(c) The efficacy of reverse transcription on immobilized substrate (B) was compared to substrate in solution (S). Libraries were constructed using total RNA from HEK293T cells. Streptavadin immobilization was omitted for solution samples during the RT step. As a control, calf intestine alkaline phosphatase (CIP) was omitted from some samples. Final PCR products of libraries were separated on a 10% TBE gel stained with SYBR Gold. Results are quantified in Figure 1e. All data from n=1 replicate shown.

(d) Periodate oxidation test with 3’ dT or 3’rU ends. Ligation was performed in solution with one donor oligonucleotide and two acceptor oligonucleotides containing 3’ terminal dT or rU. Periodate oxidation was performed as indicated, followed by ligation. Ligation products were separated on 15% TBE-urea gel and stain with SYBR Gold. All data from n=1 replicate shown.

(e) MSR-seq libraries were constructed from HEK total RNA, with (+DM) or without (-DM) demethylase treatment. The final PCR products were separated on a 10% TBE gel and stained with SYBR Gold. Bands corresponding to RT stops at known modifications are labeled; DM treatment has a strong effect on m^1A58-induced stops. Results are quantified in Figure 1f. All data from n=2 replicates shown.

(f) Schematic representation of measuring tRNA charging by sequencing in one-pot periodate oxidation and β-elimination. $NaIO_4$: sodium periodate; $Na_2B_4O_7$: sodium tetraborate.

(g) MSR-seq libraries were constructed with (+) and without (-) the one-pot β-elimination protocol from HEK total RNA. ‘Without beta-elimination' samples were separately deacylated, cleaned through a spin column, then used for library construction. By contrast, ‘with beta-elimination' samples were used directly in the first ligation reaction following chemical treatment. Final PCR products of libraries were separated on 10% TBE gel and stained with SYBR Gold. $NaIO_4$: sodium periodate, $Na_2B_4O_7$: sodium tetraborate. All data from n=1 replicate shown

(h) MSR-seq libraries were constructed from HEK total RNA using overnight RT conditions that facilitate reading through certain modifications. Final PCR products of libraries were separated on 10% TBE gel and stained with SYBR Gold. Results are quantified in Figure 1f. All data from n=3 replicates shown.

a

Sample	Spots	After trimming and >17 nt filter	% reads after trimming	Reads that map to rRNA	% of reads map to rRNA
-CMC	10740950	10406306	97%	9056121	87.0%
-CMC	17475966	17037349	97%	14825014	87.0%
+CMC	5681602	4933523	87%	4065387	82.4%
+CMC	3990200	3460452	87%	2853392	82.5%

b

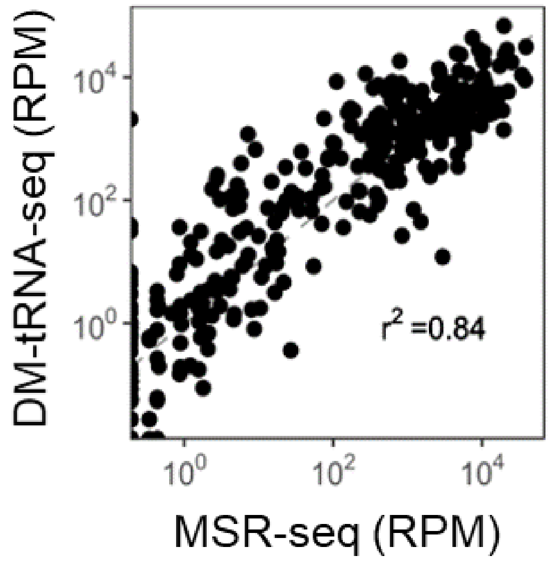

c

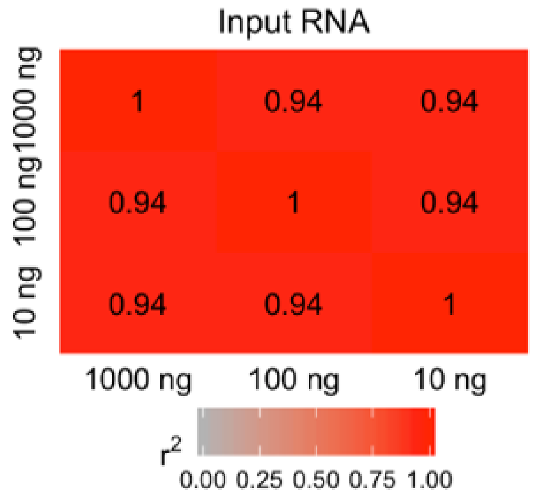

Supplementary Figure 2

Supplementary Figure 2: Chemical treatment, enzyme treatment and other results.
(a) Mapping statistics of human HEK293T total RNA samples with and without CMC treatment. >80% of the reads were mapped to human rRNA sequences.
(b) Correlation of tRNA isodecoder abundance from libraries constructed with MSR-seq with demethylase treatment compared to published DM-tRNA-seq (reference 5). RPM: reads-per-million. Sample for MSR-seq was from HEK293T total RNA; sample for DM-tRNA-seq was from gel purified HEK293T total tRNA. R^2 value corresponds to Spearmen's correlation coefficient.
(c) MSR-seq libraries were constructed from HEK total RNA with varying amounts of input material. Spearman's correlation coefficients between tRNA isodecoder abundance compared between libraries is presented.

```
LeuCAG: 5'TCTCCCCTGGAGGC(2)
SerAGA: 5'TGGGGTCTCCCCGC(2)/TGGGGTTTCCCCGC(7)
SerCGA: 5'TGGGGTCTCCCCGC(2)/TGGGGTTTCCCCGC(2)
SerTGA: 5'TGGGGTCTCCCCGC(2)/TGGGGTTTCCCCGC(2)
SerGCT: 5'TGTGCTCTGCACGC(7)/TGTGCTTTGCACGC(1)
```

Supplementary Figure 3

Supplementary Figure 3: Human tRNA variable loop sequences.
Variable loop sequences of $tRNA^{Leu}$(CAG), $tRNA^{Ser}$(AGA/CGA/TGA/GCT). The variable stem is underlined, and the C47d residue is in red. Gene copy numbers are in parenthesis. C47d and T47d $tRNA^{Ser}$ can also be distinguished in the sequences elsewhere in the tRNA body.

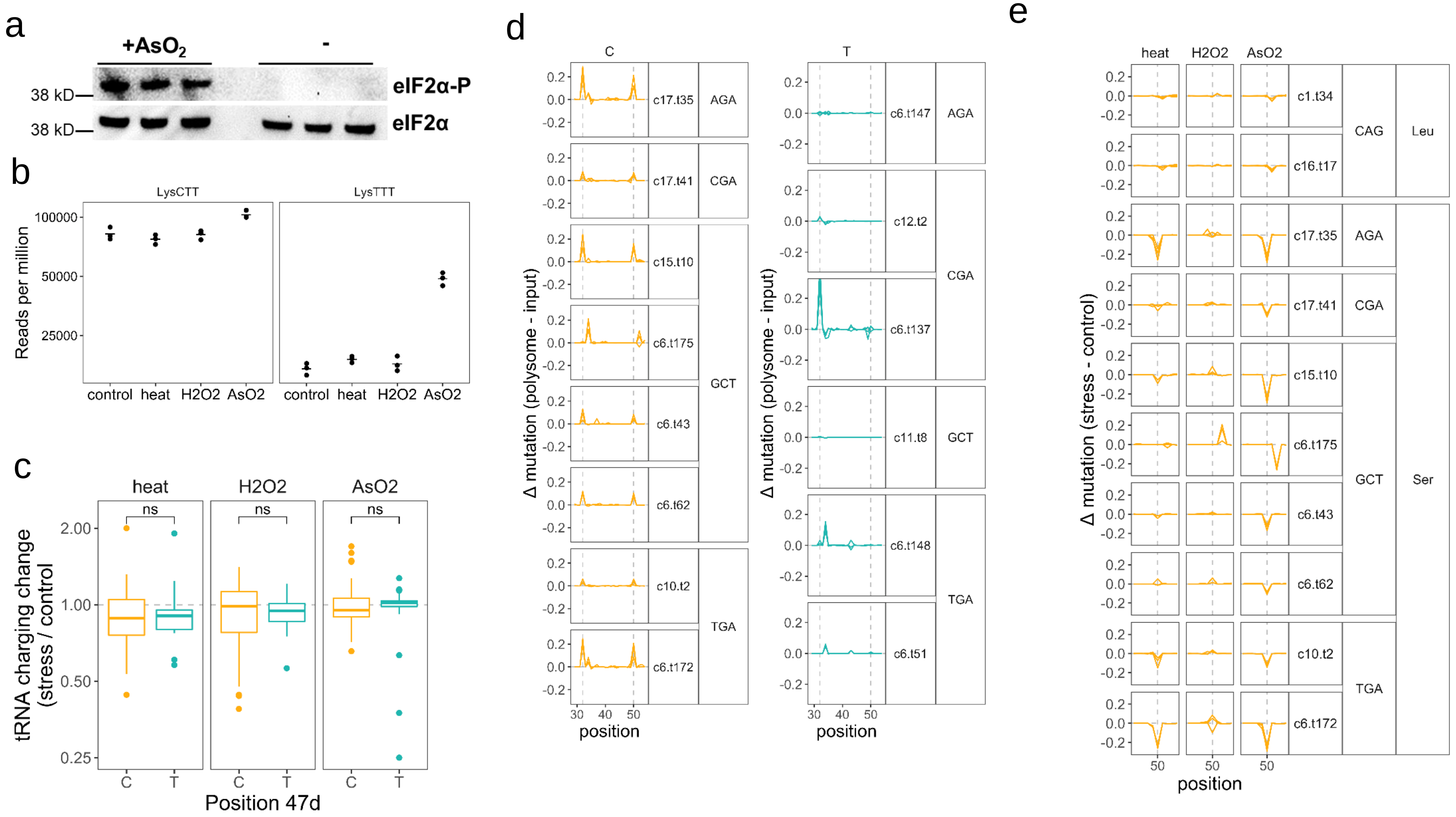

Supplementary Figure 4

Supplementary Figure 4: tRNA analysis without and with stress on the polysome.

(a) HEK cells were stressed with Arsenite (+AsO_2) or unstressed and used for sequencing. Western blot against phosphorylated eIF2α confirms a stress response mounted in treated cells, but not control cells. Blotting against total eIF2α protein is used as a loading control. Source data are provided as a Source Data file.

(b) Normalized abundance of $tRNA^{Lys}$ isoacceptor families in the polysome fraction is plotted for each stress condition. All data from n=3 biological replicates shown.

(c) Fold-change in charging (aminoacylation level) for $tRNA^{Ser}$ isodecoders in the polysome faction is plotted. Isodecoders with C at position 47d are orange, while isodecoders with T at 47d are blue. Difference between C and T isodecoders is tested with Student's two-sided T-test; *ns*: not significant. Data are shown for *n*=3 independent biological replicates of 8 and 6 C or T $tRNA^{Ser}$ isodecoders, respectively. Box and Whisker plots show median, 25th and 75th quartile, and whiskers to 1.5 times interquartile range.

(d) Differences in mutation signature near position 32 and 47d between input and polysome fractions for control (unstressed) cells are plotted for $tRNA^{Ser}$ isodecoders. C47d-$tRNA^{Ser}$ isodecoders in orange, T47d-$tRNA^{Ser}$ in blue. Results are summarized in Figure 4h.

(e) Differences in mutation signature near position 47d for $tRNA^{Leu}$ and $tRNA^{Ser}$ isodecoders in polysome fractions between stressed and control cells are plotted. Traces for each of the 3 stress replicates are shown; difference is shown as subtracted mutation fraction for each stress sample from the mean mutation fraction of control replicates. Only isodecoders with C at position 47d are shown (orange). Results are partially summarized in Figure 4i.

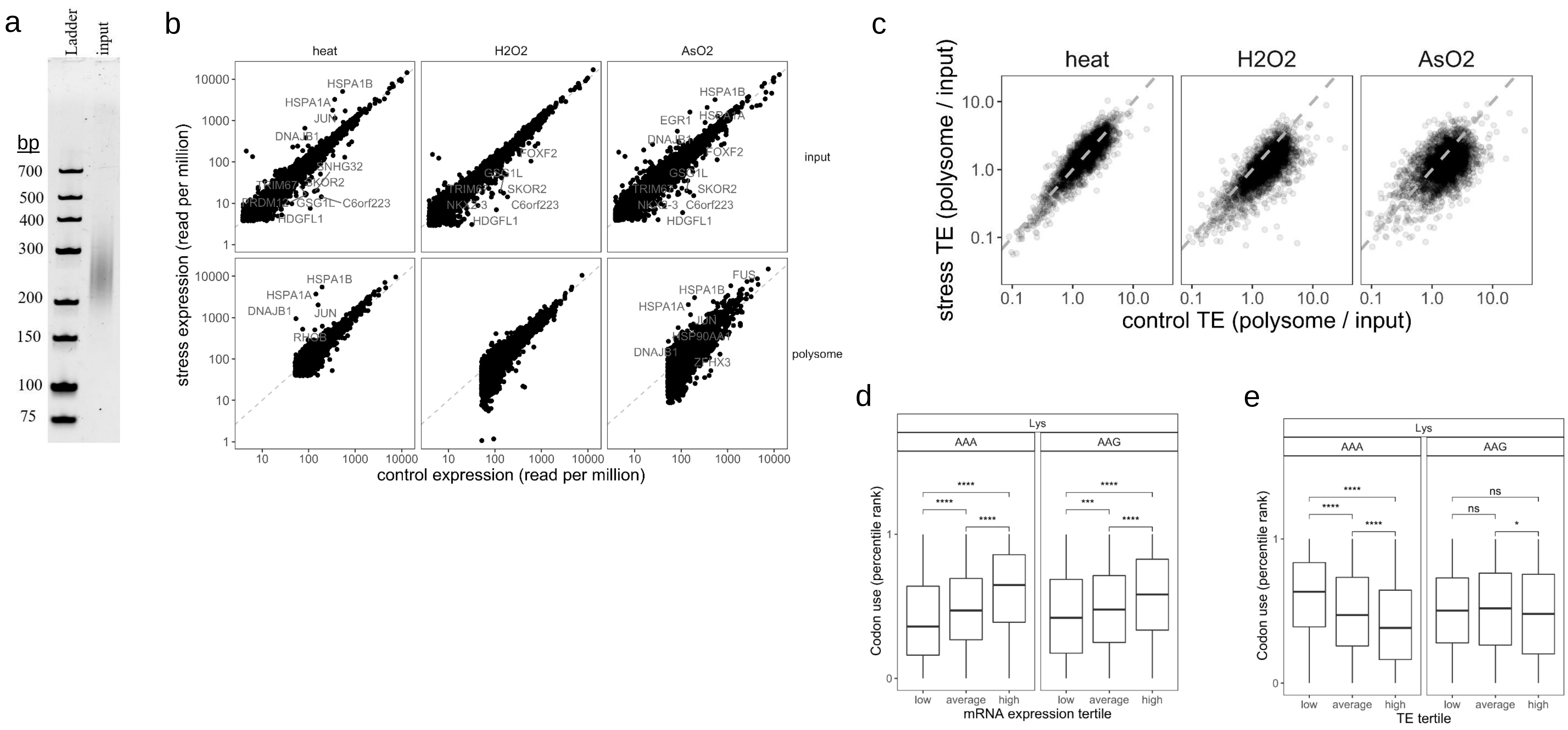

Supplementary Figure 5: legend on next page.

Supplementary Figure 5: More mRNA results.
(a) MSR-seq is compatible with input RNA of all kinds. As a demonstration, mRNA libraries were constructed from poly(A)-selected, then fragmented HEK RNA. Final PCR product of these libraries is shown; sample was separated on a 10% TBE gel and stain with SYBR Gold. Representative gel from 1 of n=3 biological replicates shown. Source data are provided as a Source Data file.
(b) Normalized abundance of mRNA is compared between stressed and unstressed cells for both total RNA and polysome fractions. Select genes with large changes in abundance are highlighted with names. Control (unstressed) abundance is plotted along the x-axis and stress-abundance is plotted on the y-axis. Input samples are plotted as the top row of graphs; polysome fractions are the bottom row.
(c) Translation efficiency (TE) is calculated as the ratio of normalized abundance in the polysome fraction to the input fraction. To visualize stress-induced changes in TE, the TE before stress for each gene is plotted along the x-axis and the TE after stress is plotted on the y-axis. Points below the diagonal indicate genes with reduced TE.
(d) Codons are not used evenly across all genes. Well detected mRNA genes were divided into tertiles based on abundance in the input fraction from unstressed cells. The frequency of codon usage for lysine AAA and AAG for each gene is used to find a percentile rank among the set of analyzed genes. Percentile rank is represented in the tertile boxplots. Both AAA and AAG codons are used more frequently in abundance genes. Statistical comparisons are done with two-sided Wilcox's test. Data are shown for n=3 biological replicates for n=1747 genes, comparing tertiles. Box and Whisker plots show median, 25th and 75th quartile, and whiskers to 1.5 times interquartile range. P-values (top to bottom) for AAA: $<2.2x10^{-16}$, $6.3x10^{-11}$, $<2.2x10^{-16}$; AAG: $2.2x10^{-16}$, $8.1x10^{-4}$, $1.3x10^{-12}$
(e) Among unstressed cells, the TE of well detected genes is calculated. Genes are divided into tertiles based on TE (low, average, high). The codon usage for lysine AAA and AAG is calculated for each gene and used to assign a percentile rank among this set of genes. Differences in percentile rank distribution between tertiles for AAA indicates that efficiently translated genes use less AAA codon than inefficiently translated genes. This same pattern is not seen for AAG. Statistical comparisons are done with two-sided Wilcoxon test. Data are shown for n=3 independent biological replicates for n=1747 genes, comparing tertiles. Box and Whisker plots show median, 25th and 75th quartile, and whiskers to 1.5 times interquartile range for AAA: $<2.2x10^{-16}$, $<2.2x10^{-16}$, $1.1x10^{-10}$; AAG: $1.9x10^{-2}$.

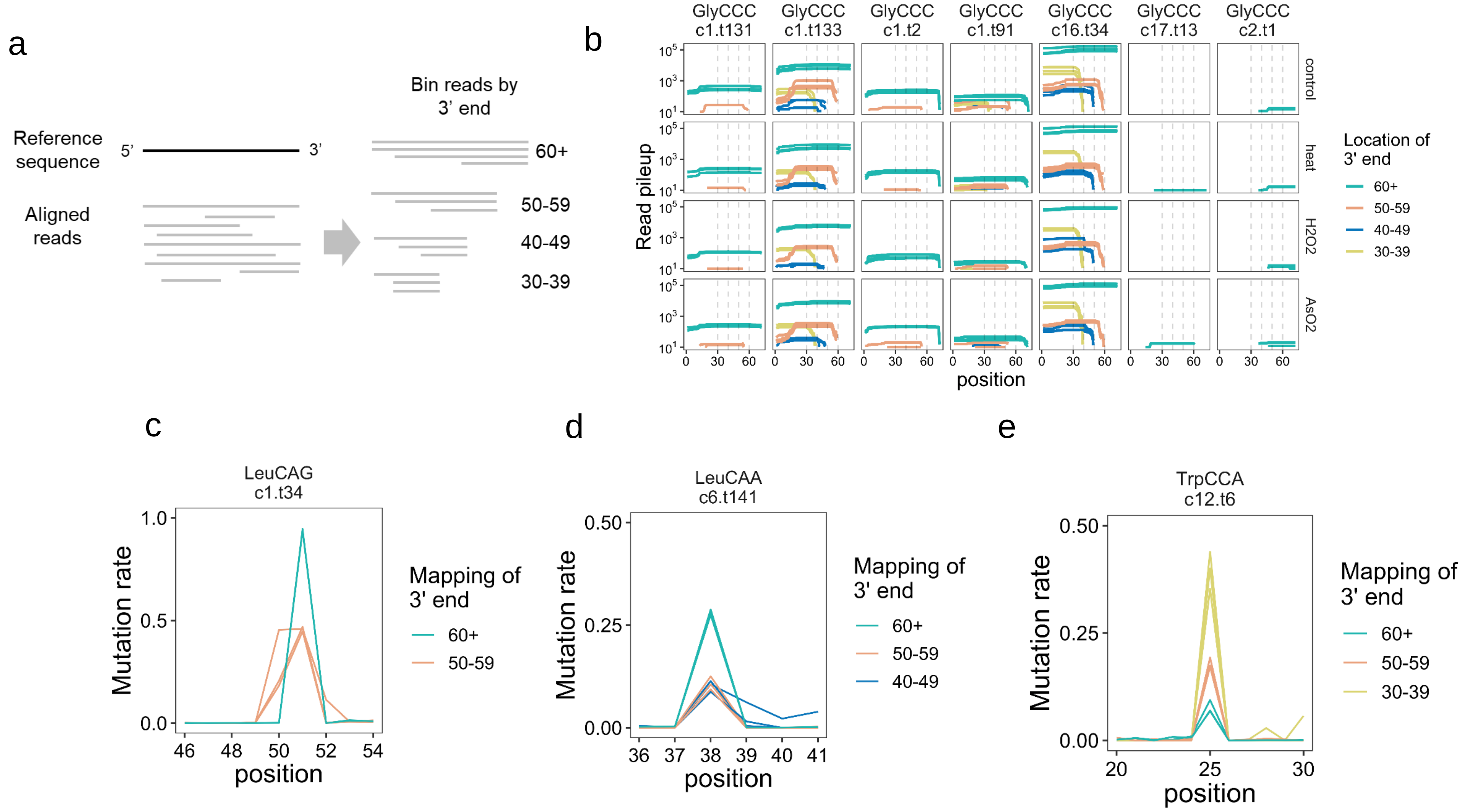

Supplementary Figure 6

Supplementary Figure 6: more tRNA and tRNA fragment (tRF) analysis.
(a) Schematics of tRF analysis strategy showing how reads are binned based on the mapped position of the 3' end of each read.
(b) Read pile-up of all tRNA^{Gly}(CCC) isodecoders, biological triplicates are shown. Reads corresponding to different fragment bins are indicated by different colors.
(c-e) Mutation signatures of the cognate full-length tRNA in blue and for tRF in pink or tan, biological triplicates (*n*=3) are shown. Distinct mutation signatures among full-length and fragmented tRNAs are highlighted for the following modification in specific isodecoders:
(c) m^{3}C47 of tRNA^{Leu}(CAG).
(d) m^{1}G37 of tRNA^{Leu}(CAA).
(e) m$^{2}_{2}$G26 of tRNA^{Trp}.
