# Supplementary material for: A multiplex platform for small RNA sequencing elucidates multifaceted tRNA stress response and translational regulation
Source: Nat Commun. 2022 May 5;13:2491. doi: 10.1038/s41467-022-30261-3 (PMC9072684; doi:10.1038/s41467-022-30261-3)
Supplement: Supplementary file 3 — Description of Additional Supplementary Files [file 41467_2022_30261_MOESM3_ESM.pdf]

Description of Additional Supplementary Files

File Name: Supplementary Data 1
Description: **tRNA abundance results of input samples at the isoacceptor level.** "Sum_count" corresponds to reads mapped to all isodecoders in each isoacceptor family. "rpm" corresponds to the ratio of reads for each isoacceptor family divided by reads for all isoacceptors in the same sample. "_1, _2, _3" corresponds to the biological replicates.

File Name: Supplementary Data 2
Description: **tRNA charging results of input samples at the isoacceptor level.** "Charging" corresponds to ratio of reads having a 3'A divided by the sum of the reads having a 3'A plus a 3'C in the same sample. "rep_1, _2, _3" corresponds to the biological replicates.

File Name: Supplementary Data 3
Description: **tRNA abundance results of polysome samples at the isoacceptor level.** "Sum_count" corresponds to reads mapped to all isodecoders in each isoacceptor family. "rpm" corresponds to the ratio of reads for each isoacceptor family divided by reads for all isoacceptors in the same sample. "_1, _2, _3" corresponds to the biological replicates.

File Name: Supplementary Data 4
Description: **tRNA charging results of polysome samples at the isoacceptor level.** "Charging" corresponds to ratio of reads having a 3'A divided by the sum of the reads having a 3'A plus a 3'C in the same sample. "rep_1, _2, _3" corresponds to the biological replicates.

File Name: Supplementary Data 5
Description: **Oligonucleotide sequences of PCR primers, barcode primers, and hairpin barcode ligation oligos.**
