# Supplementary material for: A multiplex platform for small RNA sequencing elucidates multifaceted tRNA stress response and translational regulation
Source: Nat Commun. 2022 May 5;13:2491. doi: 10.1038/s41467-022-30261-3 (PMC9072684; doi:10.1038/s41467-022-30261-3)
Supplement: Supplementary file 9 — Reporting Summary [file 41467_2022_30261_MOESM9_ESM.pdf]

nature research

Corresponding author(s): Christopher Katanski
Tao Pan

Last updated by author(s): Mar 11, 2022

Reporting Summary

Nature Research wishes to improve the reproducibility of the work that we publish. This form provides structure for consistency and transparency in reporting. For further information on Nature Research policies, see our Editorial Policies and the Editorial Policy Checklist.

Statistics

For all statistical analyses, confirm that the following items are present in the figure legend, table legend, main text, or Methods section.

n/a	Confirmed	
☐	☒	The exact sample size (*n*) for each experimental group/condition, given as a discrete number and unit of measurement
☐	☒	A statement on whether measurements were taken from distinct samples or whether the same sample was measured repeatedly
☐	☒	The statistical test(s) used AND whether they are one- or two-sided<br>*Only common tests should be described solely by name; describe more complex techniques in the Methods section.*
☐	☒	A description of all covariates tested
☐	☒	A description of any assumptions or corrections, such as tests of normality and adjustment for multiple comparisons
☐	☒	A full description of the statistical parameters including central tendency (e.g. means) or other basic estimates (e.g. regression coefficient) AND variation (e.g. standard deviation) or associated estimates of uncertainty (e.g. confidence intervals)
☐	☒	For null hypothesis testing, the test statistic (e.g. *F*, *t*, *r*) with confidence intervals, effect sizes, degrees of freedom and *P* value noted<br>*Give P values as exact values whenever suitable.*
☒	☐	For Bayesian analysis, information on the choice of priors and Markov chain Monte Carlo settings
☒	☐	For hierarchical and complex designs, identification of the appropriate level for tests and full reporting of outcomes
☒	☐	Estimates of effect sizes (e.g. Cohen's *d*, Pearson's *r*), indicating how they were calculated

Our web collection on statistics for biologists contains articles on many of the points above.

Software and code

Policy information about availability of computer code

Data collection	ImageLab v6.0
Data analysis	Paired-end reads were divided using FastX toolkit fastx_barcode_splitter version 0.0.13. Reads were mapped using bowtie2 with the "—local" flag, version 2.3.3.1-linux-x86_64. Sam files were converted to bam using samtool v 1.10 samtools view.<br>Bam files were sorted using samtools v 1.10 samtools sort. Sorted bam files were converted to pileup using IGV v 2.8.0 command "igvtools count -z 5 -w 1 -e 250 —bases". Wig files were reformatted using custom python script. Gene expression was quantified from sam files using custom python script. Data was plotted using R version 3.5.0. All custom scripts are availible at https://github.com/ckatanski/CHRIS-seq

For manuscripts utilizing custom algorithms or software that are central to the research but not yet described in published literature, software must be made available to editors and reviewers. We strongly encourage code deposition in a community repository (e.g. GitHub). See the Nature Research guidelines for submitting code & software for further information.

Data

Policy information about availability of data

All manuscripts must include a data availability statement. This statement should provide the following information, where applicable:

- Accession codes, unique identifiers, or web links for publicly available datasets
- A list of figures that have associated raw data
- A description of any restrictions on data availability

All sequencing data have been deposited to NCBI GEO under accession # GSE198441, and is now publicly available These data are associated with Figures 2-6 and supplemental figures 2-6.

Field-specific reporting

Please select the one below that is the best fit for your research. If you are not sure, read the appropriate sections before making your selection.

☒ Life sciences ☐ Behavioural & social sciences ☐ Ecological, evolutionary & environmental sciences

For a reference copy of the document with all sections, see nature.com/documents/nr-reporting-summary-flat.pdf

Life sciences study design

All studies must disclose on these points even when the disclosure is negative.

Sample size	Measurements were performed in independent biological triplicate or duplicate as validation of large effect sizes. Sequencing result validation was provided by comparing enzymatic/chemically treated samples from different groups of independent biological triplicates.
Data exclusions	No data were excluded from this analysis
Replication	Measurements were performed in triplicate or duplicate as validation of large effect sizes. Sequencing result validation was provided by comparing enzymatic/chemically treated samples from different groups of independent biological triplicates. Some results are further compared to published work on similar samples as further validation. Important results in this work are validated with non-sequencing based methods including northern blot and radio-nuclide incorporation during primer extension. All attempts at replication were successful.
Randomization	Randomization is not relevant because samples were derived from controlled defined culture conditions.
Blinding	Investigators were blinded to group allocation and data analysis.

Reporting for specific materials, systems and methods

We require information from authors about some types of materials, experimental systems and methods used in many studies. Here, indicate whether each material, system or method listed is relevant to your study. If you are not sure if a list item applies to your research, read the appropriate section before selecting a response.

Materials & experimental systems

n/a	Involved in the study	
☐	☒	Antibodies
☐	☒	Eukaryotic cell lines
☒	☐	Palaeontology and archaeology
☒	☐	Animals and other organisms
☒	☐	Human research participants
☒	☐	Clinical data
☒	☐	Dual use research of concern

Methods

n/a	Involved in the study	
☒	☐	ChIP-seq
☒	☐	Flow cytometry
☒	☐	MRI-based neuroimaging

Antibodies

Antibodies used	anti-EIF2S1; Invitrogen, AHO0802<br>anti-Phospho-EIF2S1; Invitrogen, MA5-15133<br>sheep anti-mouse IgG; Cytiva, NA931V<br>donkey anti-rabbit IgG conjugated to horseradish peroxidase; Cytiva, NA934V
Validation	Validated by manufacturer:<br>https://www.thermofisher.com/antibody/product/EIF2S1-Antibody-clone-EIF2-alpha-Monoclonal/AHO0802<br>https://www.thermofisher.com/antibody/product/Phospho-EIF2S1-Ser51-Antibody-clone-S-674-5-Monoclonal/MA5-15133<br>https://www.cytivalifesciences.com/en/us/shop/protein-analysis/blotting-and-detection/blotting-standards-and-reagents/amersham-ecl-hrp-conjugated-antibodies-p-06260 (for NA931V and NA934V)

Eukaryotic cell lines

Policy information about cell lines

Cell line source(s)	HEK293T, MCF7
Authentication	Cell lines were purchased directly from ATCC, validated by morphology.
Mycoplasma contamination	Tested negative.

Commonly misidentified lines
(See ICLAC register)

Not used
