# Supplementary material for: A multiplex platform for small RNA sequencing elucidates multifaceted tRNA stress response and translational regulation
Source: Nat Commun. 2022 May 5;13:2491. doi: 10.1038/s41467-022-30261-3 (PMC9072684; doi:10.1038/s41467-022-30261-3)
Supplement: Supplementary file 2 — Peer Review File [file 41467_2022_30261_MOESM2_ESM.pdf]

Editorial Note: This manuscript has been previously reviewed at another journal that is not operating a transparent peer review scheme. The manuscript was considered suitable for publication without further review at *Nature Communications*.
